# A brief history of insect neuropeptide and peptide hormone research

**DOI:** 10.1007/s00441-024-03936-0

**Published:** 2024-12-10

**Authors:** Dick R. Nässel

**Affiliations:** Department of Zoology, Stockholm University, S-10691 Stockholm, Sweden

**Keywords:** Neuropeptide function, Neuromodulation, Hormonal signaling, Brain circuits, Endocrine cells, *Drosophila*

## Abstract

This review briefly summarizes 50 years of research on insect neuropeptide and peptide hormone (collectively abbreviated NPH) signaling, starting with the sequencing of proctolin in 1975. The first 25 years, before the sequencing of the *Drosophila* genome, were characterized by efforts to identify novel NPHs by biochemical means, mapping of their distribution in neurons, neurosecretory cells, and endocrine cells of the intestine. Functional studies of NPHs were predominantly dealing with hormonal aspects of peptides and many employed ex vivo assays. With the annotation of the *Drosophila* genome, and more specifically of the NPHs and their receptors in *Drosophila* and other insects, a new era followed. This started with matching of NPH ligands to orphan receptors, and studies to localize NPHs with improved detection methods. Important advances were made with introduction of a rich repertoire of innovative molecular genetic approaches to localize and interfere with expression or function of NPHs and their receptors. These methods enabled cell- or circuit-specific interference with NPH signaling for in vivo assays to determine roles in behavior and physiology, imaging of neuronal activity, and analysis of connectivity in peptidergic circuits. Recent years have seen a dramatic increase in reports on the multiple functions of NPHs in development, physiology and behavior. Importantly, we can now appreciate the pleiotropic functions of NPHs, as well as the functional peptidergic “networks” where state dependent NPH signaling ensures behavioral plasticity and systemic homeostasis.

**Supplementary information:**

The online version contains supplementary material available at 10.1007/s00441-024-03936-0.

## Introduction

In 2025, it is the 50th anniversary of the sequencing of the first insect neuropeptide, proctolin (Starratt and Brown 1975). Much water has flown under the bridge since 1975, and this flow has sometimes been slow and circuitous, but occasionally leaps occurred that greatly advanced our understanding of neuropeptide signaling. A major advancement was seen at the turn of the century with the whole genome sequencing and annotation of neuropeptide precursors and receptors in the nematode worm *Caenorhabditis elegans* (Bargmann 1998; Sequencing Consortium, 1998) and fly *Drosophila melanogaster* (Adams et al. 2000; Hewes and Taghert 2001; Vanden Broeck 2001b). This made it possible to reveal the complete sets of genes encoding neuropeptide precursors and their receptors in each species. More recently, multiple methods have been devised to map the cellular distribution of neuropeptides and peptide hormones (henceforth collectively abbreviated NPHs) and their receptors. These include improved immunocytochemistry and in situ hybridization detection and confocal imaging and in the fly *Drosophila* the binary GAL4-UAS system for targeted expression of fluorescent markers (Brand and Perrimon 1993; Duffy 2002). The recent 10–15 years have been especially rewarding with the application of innovative molecular genetic tools in NPH research in *Drosophila*. Fortunately, many other insects also became genetically tractable due to the development of RNA-interference and CRISPR/Cas9 technology. Some of the most striking findings over the years since 1975 are the discovery that a very large number of NPHs exists in each species and that NPHs display extensive functional diversity [see (Jékely et al. 2018; Nässel et al. 2019; Nässel and Zandawala 2019; Ragionieri et al. 2021; Schoofs et al. 2017)]. Furthermore, it was shown that single NPHs can have multiple pleiotropic actions [see (Nässel et al. 2019; Nässel and Zandawala 2019; Schoofs et al. 2017). Genomic data from multiple animal species have revealed that neuropeptide signaling arose early in animal evolution, and numerous aspects of structure and function of NPHs and their receptors are evolutionarily conserved among metazoan animals (Beets et al. 2023; Elphick et al. 2018; Hewes and Taghert 2001; Jékely 2013; Jékely et al. 2018; Mirabeau and Joly 2013; Vanden Broeck 2001b). In other words, a fly, a mouse, and a human display many similarities in NPH signaling pathways, although the detailed mechanisms of action may differ. It is clear that NPHs constitute the most ubiquitous and diverse class of signaling substances both structurally and functionally. NPHs act at various levels in the central and peripheral nervous system (CNS and PNS) both as primary messengers, as neuromodulators, and in interorgan signaling as hormones (Nässel and Zandawala 2019; Orchard and Lange 2024; Rajan and Perrimon 2011; Schoofs et al. 2017). Neuropeptides can be produced in most major types of neurons, including sensory cells/neurons, interneurons, and motoneurons. Hormonal peptides are produced both in neurosecretory cells of the CNS and in peripheral secretory (neurosecretory and endocrine) cells, and thus, signaling can go both from the CNS to periphery and vice versa (Nässel and Zandawala 2019; Orchard and Lange 2024; Schoofs et al. 2017). Now we know that the functionally pleiotropic NPHs in insects regulate numerous important aspects of physiology and behavior throughout the life cycle (Nässel and Zandawala 2019; Orchard and Lange 2024; Schoofs et al. 2017). Thus, most aspects of daily life, even of a fly, involve actions of NPHs at one level or another.

Insects appeared rather late in neuropeptide research, compared to vertebrates, but progress has been rapid, and this review describes the road taken to get where we are today. I will first describe the early struggle to get a handle of the identities of NPHs in insects (NPH discovery and early functional studies). Then I try to summarize how this lead up to the very expansive research it is today. Due to the large increase in insect NPH studies over the last 10–15 years, I can only briefly summarize the more recent findings here and provide examples that illustrate some of the major achievements. Furthermore, I discuss a number of questions that still remain open in insect NPH research. However, first I provide a brief history of the detection, identification, tissue mapping, and early functional analysis of NPHs in insects.^1^

### Neuropeptides: the early days of discovery

Insects have been utilized in endocrinology since 1917 starting with the pioneering experiments by Stefan Kopeć who discovered a head-derived hormone that induces pupariation in larvae of a lepidopteran insect, *Lymantria dispar* (Kopeć, 1917, 1922), later known as prothoracicotropic hormone (PTTH) a peptide hormone [see (Ishizaki and Suzuki 1994)]. The recognition of specific neurosecretory cells (NSCs) by Ernst and Berta Scharrer in the 1940s suggested that neurons in the brain of both vertebrates and insects can be the source of hormones, similar to gland cells in other tissues [see (Scharrer 1987; Scharrer and Scharrer 1963)]. These early studies also indicated analogies between the hypothalamus-pituitary axis and the insect pars intercerebralis-corpora cardiaca/corpora allata axis [see also (Hartenstein 2006; Nässel and Zandawala 2020; Orchard and Lange 2024; Raabe 1989)]. Whereas the pituitary peptide hormones oxytocin and vasopressin were identified and sequenced already in the 1950s (Davoll et al. 1951; Turner et al. 1951) and hypothalamic ones such as thyrotropin-releasing hormone (TRH) and gonadotropin-releasing hormone (GnRH) in the 1960s (Guillemin et al. 1964; Schally et al. 1966), insect NPHs were sequenced much later. An important discovery made in mammals was that NPHs are not only produced by NSCs, but also by regular neurons, some that act to regulate hormone release, others that are interneurons or sensory neurons [see (Guillemin 1978; Hökfelt et al. 1980, 1975)]. Furthermore, it was demonstrated that also endocrine cells of the intestine (enteroendocrine cells, EECs) produce peptides [reviewed in (Mutt 1982, 1990)], and some of these gut peptides are also produced by neurons in the brain referred to as “brain-gut peptides.” Actually, the first *bona fide* neuropeptide identified, substance P, was originally isolated both from brain and intestinal tissue (Von Euler and Gaddum 1931). Thus, at the time, insect research on NPHs entered a more active phase in the 1970s; it was already known that in mammals NPHs play functional roles in the CNS and PNS as well as in the intestinal tract and as circulating hormones.

The first insect neuropeptide to be identified and sequenced was proctolin in 1975 (Starratt and Brown 1975), followed by the peptide hormone adipokinetic hormone, AKH (Stone et al. 1976) a year later. It is noteworthy that although proctolin is a pentapeptide (RYLPT) in those days it required 125,000 cockroaches (125 kg fresh weight) to purify enough material for sequencing. Interestingly, it was shown that AKH displays sequence similarities to the crustacean NPH red pigment concentrating hormone (RPCH), which had been sequenced already in 1972 (Fernlund and Josefsson 1972). This was an early indication that there might be “families” of related neuropeptides in different groups of invertebrates. These peptides remained the only sequenced endogenous NPHs from insects for several years. Antisera were raised to these peptides enabling immunohistochemical localization of AKH and proctolin (Bishop and O'Shea 1982; Eckert et al. 1981; Schooneveld et al. 1983). This meant that for the first time we could see the cellular expression of native NPHs in insects. AKH was found exclusively in the NSCs of the corpora cardiaca (Schooneveld et al. 1983), whereas proctolin turned out to have a widespread distribution in interneurons and specific motoneurons (Bishop and O'Shea 1982; Eckert et al. 1981; O'Shea and Bishop 1982).

In the meantime, there were a few years of exploration of possible presence of NPHs in insects by immunohistochemistry with antisera raised against vertebrate NPHs. This early exploration served several purposes: (1) as a relatively quick screen for putative endogenous neuropeptides based on cross reactivity with the heterologous antisera, (2) as a neuroanatomical tool to delineate neuronal systems (identifiable neurons) that were specified by a particular molecular phenotype (chemical neuroanatomy), (3) to enable analysis of the development of specific identified neurons, and (4) to use chemical anatomy to delineate neuronal systems in different insect species, thereby determining to what extent identified neurons are phylogenetically conserved. A selection of such early studies exploring immunolabeling with heterologous antisera can be seen in the following papers (Duve and Thorpe 1979; El-Salhy et al. 1980; Kramer et al. 1977; Rémy et al. 1977). Some laboratories took the immunohistochemistry with heterologous antisera a step further and attempted isolation and structure determination by assaying purification steps with radioimmunoassays. Several of these were labor intensive and did not lead to full structure determination, but suggested presence of related peptides in blowflies: for instance insulin (Duve et al. 1979) and pancreatic polypeptide/neuropeptide Y (Duve et al. 1982). A number of years later, these were identified in for instance *Drosophila* as insulin-like peptides (Brogiolo et al. 2001) and neuropeptide F (Brown et al. 1999), respectively. The insulin-like peptide bombyxin was identified in the silk moth *Bombyx mori* already in 1989 based on its hormonal action in development (Iwami et al. 1989).

In the mid-1980s, there were strong efforts aimed at isolating and sequencing peptides that affect the activity of visceral muscle of the cockroach *Leucophaea maderae* or the locust *Locusta migratoria*. Hence, during purification of peptides from the brains of these species, fractions were tested in vitro for myoactivity. Most cockroach work was done in the laboratory of Mark Holman and Ronald Nachman in College Station, Texas, and the locust peptide work in the laboratory of Arnold De Loof and Liliane Schoofs in Leuven, Belgium. These efforts were very successful, and numerous NPHs were identified [reviewed in (Holman et al. 1991, 1990; Schoofs et al. 1993, 1997)], which have later been discovered also in other insects or other invertebrates. Table 1 shows NPHs isolated in early studies (until 1993), both using the muscle assays and other assays (see below). Examples of NPHs originating from these early studies that have been intensely investigated in recent years in insects, including *Drosophila*, are insulin-like peptides (ILPs), pigment-dispersing factor (PDF), tachykinins (TK), leucokinins (LK), neuropeptide F (NPF), short neuropeptide F (sNPF), pyrokinins (PK), and sulfakinins (SK). We will get back to these NPHs later for further functional aspects.

**Table 1:** Neuropeptides and peptide hormones known in 1993

Peptide^1^	Acronym^2^	First sequenced from	Reference
Adipokinetic hormone	Lom-AKH (AKH)	*Locusta* (locust)	(Stone et al. 1976)
Allatostatin	Mas-AS (AstC)	*Manduca* (moth)	(Kramer et al. 1991)
Allatostatin	Dip-AS (AstA)	*Diploptera* (cockroach)	(Pratt et al. 1989)
Allatotropin	Mas-AT (AT) [nd]	*Manduca* (moth)	(Kataoka et al. 1989a)
Bombyxin	Insulin-like peptide (ILP)	*Bombyx* (moth)	(Nagasawa et al. 1986)
Cardioactive peptide	CAP_2b_ (CAPA)	*Manduca* (moth)	(Huesmann et al. 1995)^3^
Cardioactive peptide	CAP_2a_ (CCAP)	*Manduca* (moth)	(Cheung et al. 1992)
Corazonin	CRZ	*Periplaneta* (cockroach)	(Veenstra 1989)
Diapause hormone	Bom-DH ^4^ [nd]	*Bombyx* (moth)	(Sato et al. 1993)
Diuretic hormone	Mas-DP, CRF-like DH, (DH44)	*Manduca* (moth)	(Kataoka et al. 1989b)
Eclosion hormone	Mas-EH (EH)	*Manduca* (moth)	(Kataoka et al. 1987)
FMRFamide-like	Head peptide (HP, sNPF)	*Aedes* (mosquito)	(Matsumoto et al. 1989)
FMRFamide-like	dFMRFamide	*Drosophila* (fly)	(Nambu et al. 1988; Schneider and Taghert 1988)
FMRFamide-like	Myosuppressin (MS, DMS)	*Leucophaea* (cockroach)	(Holman et al. 1986a, b, c)
Leucokinins	LK	*Leucophaea* (cockroach)	(Holman et al. 1986a)
Myoinhibitory peptide	Lom-MIP (MIP, AstB)	*Locusta* (locust)	(Schoofs et al. 1991)
Neuroparsin	NP [nd]	*Locusta* (locust)	(Girardie et al. 1989)
PBAN ^5^	PBAN [nd]	*Bombyx, Heliotis* (moths)	(Raina et al. 1989; Sato et al. 1993)
Pigment disp. factor	PDF	*Romalea* (locust)	(Rao et al. 1987)
Proctolin	Proct	*Periplaneta* (cockroach)	(Starratt and Brown 1975)
PTTH ^6^	PTTH	*Bombyx* (moth)	(Kataoka et al. 1991)
Pyrokinins	Lem-PK (PK)	*Leucophaea* (cockroach)	(Holman et al. 1986b)
Sulfakinins	Lem-SK (SK, DSK)	*Leucophaea* (cockroach)	(Nachman et al. 1986)
Tachykinins	Lom-TK (TRP, TK)	*Locusta* (locust)	(Schoofs et al. 1990)
Vasopressin-like	VPL (inotocin) [nd]	*Locusta* (locust)	(Proux et al. 1987)

^1^The first general annotation of peptide (as used in 1993)

^2^Acronyms given to first characterized peptide. The acronyms in brackets are the ones currently used in *Drosophila*. If no such acronym is given the peptide either does not exist in *Drosophila* [nd] or the original acronym is used

^3^Available as abstract in 1992 (Loi et al. 1992)

^4^A pyrokinin-like peptide from PBAN gene (see (Sato et al. 1993))

^5^Pheromone biosynthesis activating neuropeptide

^6^Prothoracicotropic hormone

There were also other laboratories where individual NPHs from insects were identified biochemically (see Table 1), but in the early 1980s, the number of known NPHs was still relatively small. In a review in Cell and Tissue Research in 1993 (Nässel 1993b), I listed 26 distinctly different neuropeptides that were known in insects at the time (see Table 1). Each of these 26 main types is today known to be encoded by a separate gene. Several NPHs isolated in the 1980s were found in several closely related isoforms in the same species. As an example, eight LKs (LK-I–LK-VIII) were isolated from the cockroach *L. maderae* (Holman et al. 1987), and a similar peptide was demonstrated in locusts (Lom-K) (Schoofs et al. 1992a). Thus, if counting isoforms within and between species, the total number of sequenced peptides in insects was much larger in 1993 than seen in Table 1. By the end of the 1990s, identified NPHs were more numerous [see (Gäde 1997; Schoofs et al. 1997)]. To get a more recent overview of the diversity in NPHs in a very large number of insect species, the reader is referred to the DINeR database (https://www.neurostresspep.eu/diner) (Yeoh et al. 2017). Fortuitously, the grouping of peptides in Table 1 is mostly valid even today. I mention this because at the time very few NPH precursor genes had been cloned from insects [see (Bogerd et al. 1995; Davis et al. 1992; Hekimi et al. 1989; Iwami et al. 1989; Nambu et al. 1988; Nichols et al. 1988; Noyes and Schaffer 1990; Sato et al. 1993; Schneider and Taghert 1988)], and it was not clear how most neuropeptides and neuropeptide isoforms were encoded on genes. From the available data at the time we knew that the bombyxin precursor encoded one peptide (Iwami et al. 1989), the FMRFamide precursor could give rise to 8 different related peptides (isoforms or paracopies) and that of the sulfakinin two related peptides (Nambu et al. 1988; Nichols et al. 1988; Schneider and Taghert 1988). The *Bombyx* gene encoding diapause hormone also produces pheromone biosynthesis activating neuropeptide (PBAN) and three other pyrokinin-like peptides (Sato et al. 1993). In contrast, the three known forms of AKH (with N-terminally similar sequences) in the locust are encoded on three separate genes (Bogerd et al. 1995). Many sequence-related peptides (primarily carboxyterminal similarities) were known that could be encoded either on one or multiple precursors: for example, the multiple FMRFamide-related peptides that were not encoded on the dFMRFamide precursor [NPHs today known as neuropeptide F (NPF), head peptides or short Neuropeptide F (sNPF), sulfakinins (SK), and myosuppressin (MS)]. Now we know that these FMRFamide-related peptides are encoded on at least six different genes in a species [see (Nässel and Zandawala 2019)]. It was actually only when the *D. melanogaster* genome was sequenced (Adams et al. 2000) and a multitude of genes associated with NPH precursors were annotated by bioinformatics (Hewes and Taghert 2001; Vanden Broeck 2001b) that it was possible to associate all the biochemically identified neuropeptides with precursors. This also enabled the establishment of relations between neuropeptides in other species like honey bees and mosquitos [see (Hauser et al. 2006; Riehle et al. 2002)]. Furthermore, one could now get a first handle on the total number of NPHs in an organism. Today, we know that there are between 50 and 70 NPH precursors in different species of insects [see (Nässel and Zandawala 2019; Ragionieri et al. 2021)]. A recent study demonstrates that if one includes alternative splicing of genes, the number of NPH precursor transcripts in the locust *Schistocerca gregaria* is not less than 81 (Ragionieri et al. 2021).

### Mapping the distribution of NPHs in early studies

Before the late 1970s, insect neurosecretory cells (NSCs) were identified by histochemical staining techniques using dyes such as chrome-hematoxylin-phloxine, paraldehyde fuchsin, or azan [see (Raabe 1989; Rowell 1976)]. There were at the time no clues to the identity of the hormones produced, although some studies made extirpations of NSCs to determine physiological effects, or dissected out groups of NSCs and used for extract to inject in other insects to assay function. As an example, extract from dissected median neurosecretory cells (MNCs) from the blowfly *Calliphora erythrocephala* had a hypoglycemic and hypotrehalosemic effect when injected in other flies (Duve 1978). Today, we know that in *Drosophila* a subpopulation of these neurons produce insulin-like peptides (ILPs) (Brogiolo et al. 2001; Cao and Brown 2001) and regulate carbohydrates (Rulifson et al. 2002).

This section deals with the years between late 1970s and mid-1990s during which immunohistochemistry was employed on insect tissue with a slowly increasing number of antisera. It is of note that when mapping of the distribution of NPHs in insects started in the late 1970s and early 1980s it was mainly based on using antisera raised to NPHs from mammals or other vertebrates (e.g., insulin, cholecystokinin, pancreatic polypeptide, vasopressin, substance P, enkephalins, and others). These earliest studies investigated the brains of the moth *Manduca sexta* (Kramer et al. 1977), the fly *Eristalis aeneus* (El-Salhy et al. 1980), and the stick insect *Clitumnus extradentatus* (also known as *Medauroidea extradentatus*) (Rémy et al. 1977). Some further papers were published the following years on for instance cockroaches (Hansen et al. 1990, 1987; Scharrer 1987) and blowflies (Duve and Thorpe 1979, 1982, 1988) that mapped the distribution of cells reacting with antisera to enkephalin, cholecystokinin, pancreatic polypeptide and insulin. Not surprisingly, the first studies of the distribution of endogenous NPHs were on proctolin (Bishop and O'Shea 1982; Eckert et al. 1981) and AKH (Schooneveld et al. 1983). Proctolin was detected in numerous interneurons and motoneurons, whereas AKH was only seen in glandular cells of the corpora cardiaca (when a specific N-terminus-directed antiserum was used). In most cases, these earlier mapping studies were somewhat superficial and indicated the location of the labeled cell bodies and some diffuse immunolabeling in brain neuropil but rarely revealed the entire extent of the neurons or neurosecretory cells expressing them. This was due to the methods used at the time (fixation method, paraffin sections, reagents for antiserum detection, and so on), and confocal microscopy was not yet available for optimal image capture. Nevertheless, later in the 1980s and early 1990s, there was an increased number of investigations charting distribution of endogenous insect NPHs such as eclosion hormone (Truman and Copenhaver 1989), FMRFamide (Veenstra and Schooneveld 1984; White et al. 1986b), bombyxin (Mizoguchi et al. 1987), *Manduca* diuretic hormone (Veenstra and Hagedorn 1991), allatostatin A (Yoon and Stay 1995), corazonin (Veenstra and Davis 1993), leucokinin (Nässel et al. 1992), tachykinin (Nässel 1993a), CCAP (Dircksen et al. 1991), PTTH (Mizoguchi et al. 1990; O'Brien et al. 1988), pyrokinin (Schoofs et al. 1992b), PBAN (Kingan et al. 1992), and pigment-dispersing factor (PDF) (Homberg et al. 1991; Nässel et al. 1991). One study investigated the *Drosophila* CNS with antisera to several NPHs from other insect species (bombyxin from *Bombyx*, and the *Manduca* peptides PTTH, allatostatin-C, and *Manduca* diuretic hormone) (Žitňan et al. 1993b).

One picture emerging in the late 1980s was that NPHs are distributed in relatively sparse (and stereotypic) populations of neurons in the CNS and that many of the peptidergic neurons are either individually identifiable (as bilateral pairs) or located in identifiable clusters [see (Anderson et al. 1988; Bishop and O'Shea 1982; Nässel et al. 1988a; Veenstra et al. 1984; Veenstra and Schooneveld 1984; White et al. 1986b)]. The concept of identifiable neurons includes that they can be recognized from specimen to specimen of one species, but also in other related species [see (Rowell 1976)]. One of the early examples of identifiable neurons are the neurosecretory cells of the insect brain and VNC (Rowell 1976) and exploration of NPHs in these cell groups commenced early on as shown below.

To summarize the immunohistochemical data obtained up to this point (until 1995), prior to use of improved whole-mount techniques and confocal imaging for optimal resolution, it was found that the hormonal peptides AKH, bombyxin (ILP), eclosion hormone, *Manduca* diuretic hormone, and PTTH are produced solely by endocrine or neurosecretory cells (NSCs) (Copenhaver and Truman 1986; Mizoguchi et al. 1987; O'Brien et al. 1988; Veenstra and Hagedorn 1991). Several other NPHs, like crustacean cardioactive peptide (CCAP), FMRFamide, LK, and TK were detected in both NSCs and interneurons (Dircksen et al. 1991; Nässel 1993a; Nässel et al. 1992; White et al. 1986b), and finally, PDF was detected in brain interneurons and efferent neurons of the VNC (Homberg et al. 1991; Nässel et al. 1993, 1991). Proctolin was additionally described in motoneurons and their axon terminations in neuromuscular junctions (Adams and O'Shea 1983; O'Shea and Bishop 1982).

**Fig. 1:**
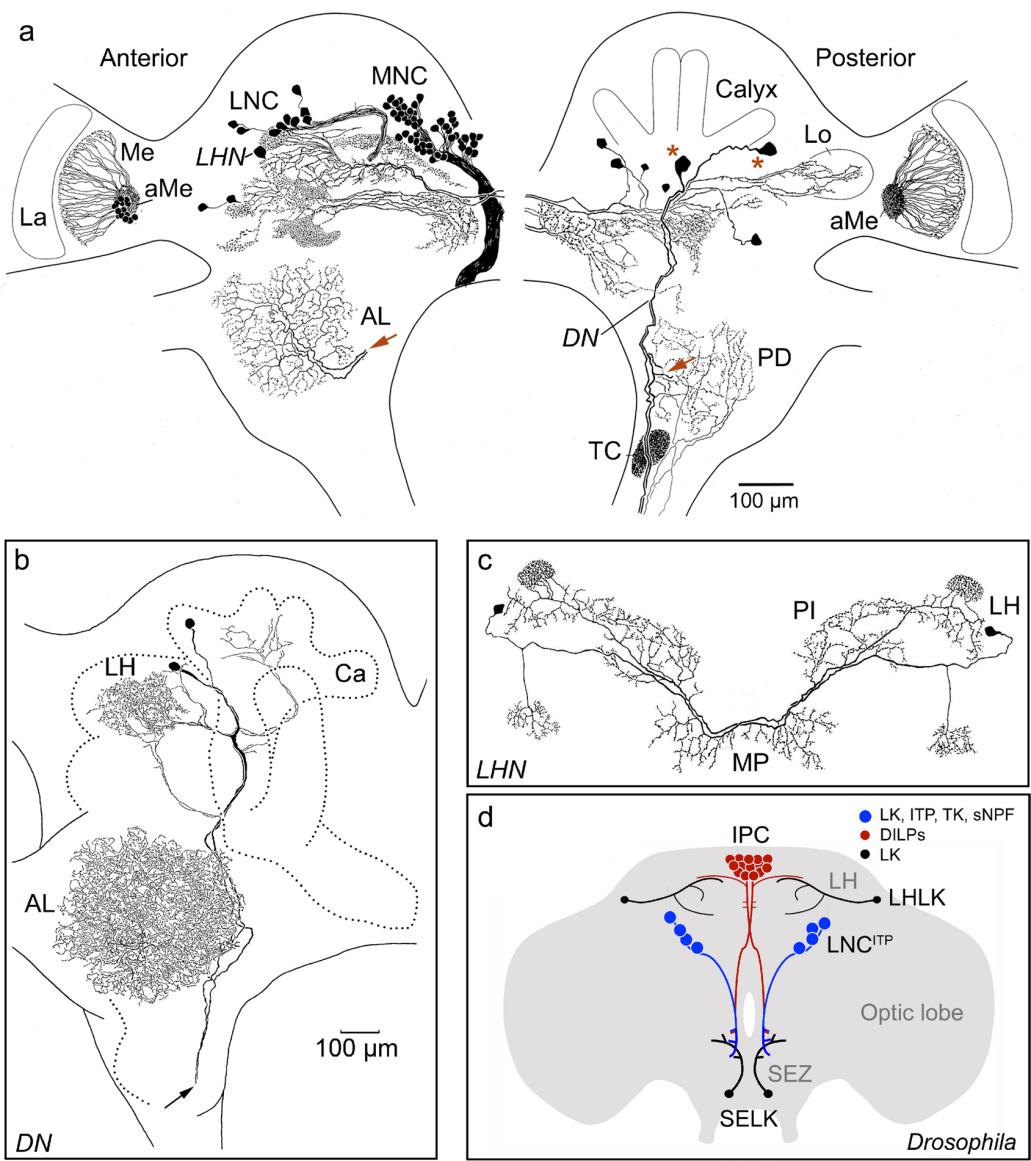
Leucokinin (LK)-expressing neurons in the brain of the cockroach *Leucophaea maderae* (*Rhyparobia maderae*) and fly *Drosophila* illustrate differences in cell number and identifiable neurons. **a** Anterior part of the cockroach brain is shown to the left and posterior to the right. The brain contains about 160 LK neurons, all with cell bodies in the protocerebrum. There are LK expressing lateral (LNC) and median neurosecretory cell groups (MNC). A pair or descending neurons (DN) in each hemisphere (cell bodies at red asterisks) send axons to the VNC and have collateral branches arborizing in the antennal lobe (red arrows show where collaterals are). A set of LK neurons have processes in the accessory medulla (aMe) and medulla (Me). A neuron in each hemisphere (*LHN*) has arborizations similar to the LHLKs (lateral horn LK neurons) in *Drosophila* (see panel **d**). Other abbreviations: La, lamina; TC, tritocerebral neuropil; PD, posterior deutocerebrum, Lo, lobula. **b** Detailed tracing of the descending neurons (DN) with branches in the antennal lobe (AL), lateral horn (LH), and calyx (Ca) of mushroom body and axons (arrow) running through the circumesophageal connectives to the ventral nerve cord. **c** A pair of cockroach LK neurons (*LHN*) with branches in lateral horn (LH), superior median protocerebrum (PI), and median protocerebrum, resembling LHLKs in *Drosophila* (see panel **d**). **d** Schematic depiction of the four main LK neurons (black) in the *Drosophila* brain. LHLKs are located in the lateral horn (LH) and branch extensively in dorsolateral protocerebrum and contact the insulin-producing cells (IPC). SELKs (subesophageal LK neurons) are descending neurons with extensive branches in the subesophageal zone (SEZ). The LNC^ITP^ are lateral neurosecretory cells that only express LK in small and variable amounts in the adult and are known to coexpress ion transport peptide (ITP), tachykinins (TK), and short neuropeptide F (sNPF). The insulin-producing cells are also shown since they are known to be regulated by the LHLKs (Yurgel et al. 2019). This figure is slightly altered from (Nässel and Wu 2021). Originals for panels **a** and **c** are from (Nässel et al. 1992) and **b** is from (Nässel and Homberg 2006) (the tracing by Uwe Homberg), all with permission

Some of the studies of further types of NPHs (published in the 1990s), reconstructed the anatomy of peptidergic neurons in more detail and attention was paid to the diversity of labeled neurons. This was by using peroxidase-mediated detection of immunolabeling and camera lucida drawings of neurons from tissue sections (cryostat or vibratome) or wholemounts (see Fig. 1). Thus, immunolabeling with antisera to, e.g., CCAP, corazonin, LK, PDF, TK, and FMRFamide was used for more detailed description of different peptidergic neuron types in the insect CNS (Cantera et al. 1994; Dircksen et al. 1991; Homberg et al. 1990, 1991; Lundquist et al. 1994; Lundquist and Nässel 1990; Nässel et al. 1988b, 1993, 1991). It became apparent that some NPHs are abundant and widespread in the CNS (and other tissues), whereas others display a sparse occurrence. Interestingly this can vary between insect species for a given NPH. For example, LK is present in four neurons of the *Drosophila* brain, but in the cockroach *Rhyparobia maderae* (formerly *Leucophaea maderae*), there are 160 neurons and in the Locust *Locusta migratoria* about 140 [reviewed in (Nässel and Wu 2021)] (Fig. 1). Already in these early studies, it was revealed that most NPHs display unique distribution patterns in neurons and NSCs, as well as occasionally in gut EECs or other peripheral cells. These features are still valid today when most types of NPHs have been mapped in *Drosophila* (and some other insects). Examples of sparse and widespread distributions in *Drosophila* are SIFamide which is only produced by four brain neurons (Terhzaz et al. 2007) and sNPF that is produced by more than 1000 (including a subpopulation of intrinsic mushroom body neurons (Johard et al. 2008). It also became clear that different insect NPHs can be produced by all the major types of neurons (including sensory cells and motoneurons), NSCs, EECs, adipocytes, gland cells, muscle cells, and cells in the reproductive organs [see (Nässel and Zandawala 2019)]. Peptidergic interneurons either display local branching of their processes (e.g., olfactory sensory neurons, Kenyon cells of mushroom bodies, antennal lobe local neurons, and some clock neurons) or widespread branching and extended axons (e.g., projection neurons, wide field neurons like SIFamide neurons, and some clock neurons).

**Fig. 2:**
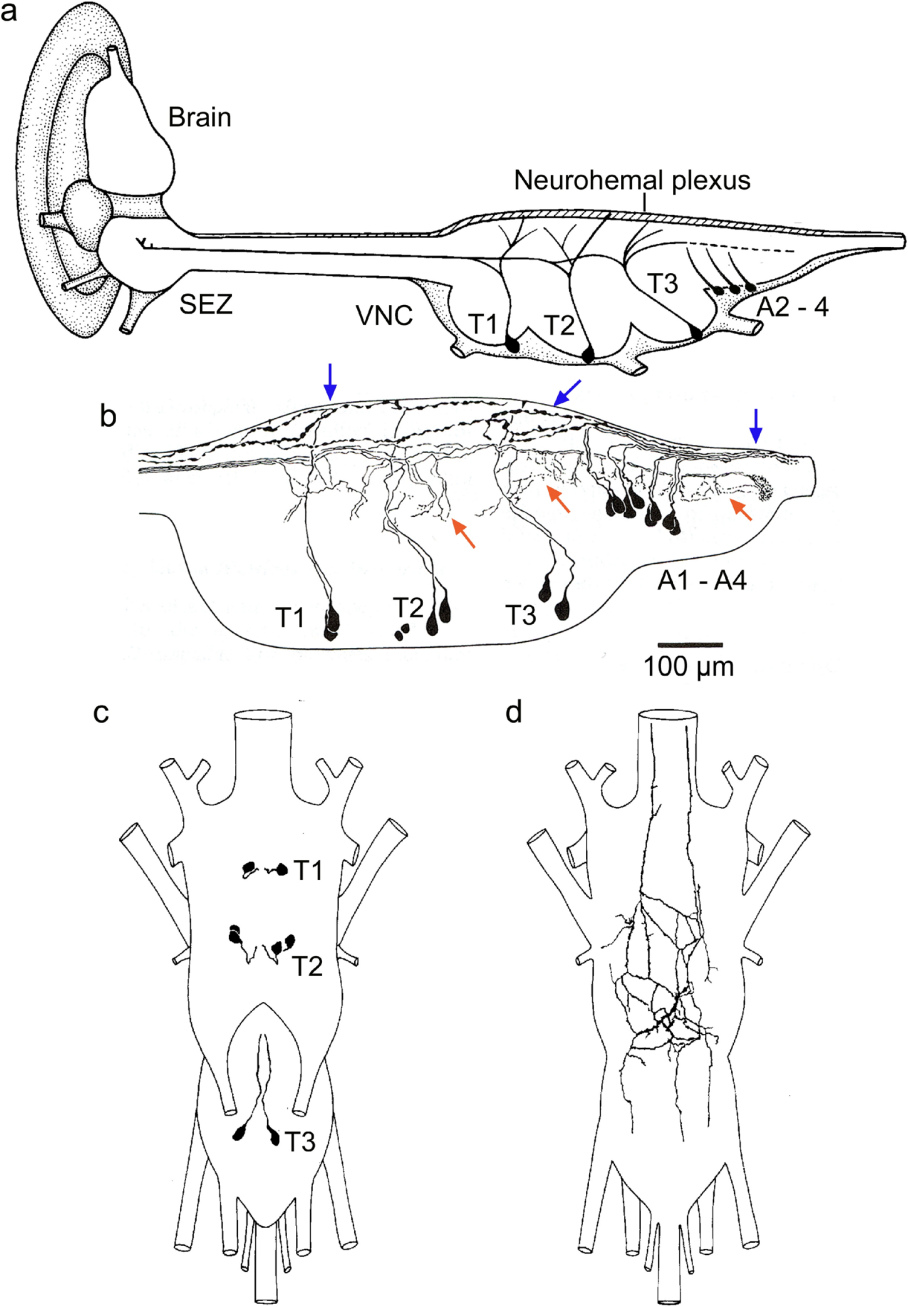
Neurosecretory cells in the ventral nerve cord send axon terminations to a neurohemal plexus in the dorsal neural sheath. **a** Schematic of brain, subesophageal zone (SEZ) and ventral nerve cord (VNC) in a blowfly, *Calliphora vomitoria*. Neurosecretory cells in the three thoracic (T1–T3) and three abdominal neuromeres (A2–A4) send axons to terminate in a plexus in the dorsal neuronal sheath of the VNC. The thoracic NSCs produce FMRFamide and the abdominal ones CAPA. **b** Tracing of neurons in the blowfly VNC reacting with antiserum to the mollusk peptide myomodulin, that apparently cross-reacts with epitopes in neurons expressing FMRFamide (T1–T3) and CAPA (A1–A4). Note that the thoracic neurons (Tv neurons) form branches inside the VNC (red arrows) and axon terminations in the dorsal neural sheath (blue arrows). **c, d** The FMRFamide expressing Tv neurons in the VNC of *Drosophila* innervate the neurohemal area in the dorsal neural sheath. A ventral view with cell bodies in **c** and a dorsal view plexus in **d**. Panel **a** is from (Lundquist and Nässel 1990), **b** from (Nässel 2002), and **c**, **d** from (Lundquist and Nässel 1990), all with permission

**Fig. 3:**
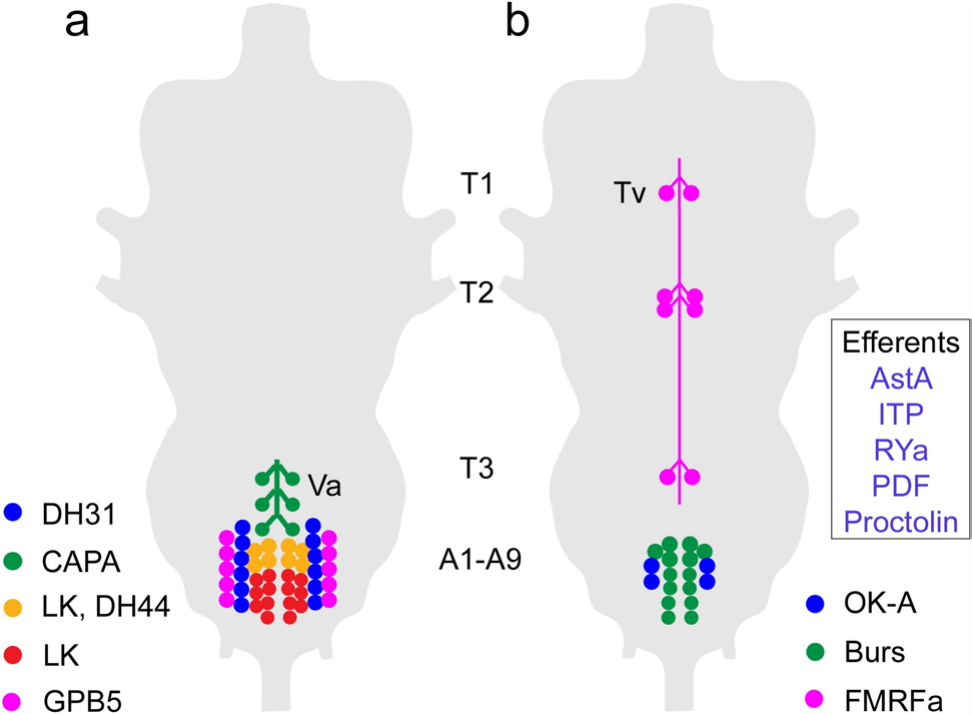
Schemes depicting neurosecretory cells and efferent neurons in the adult ventral nerve cord of *Drosophila*. Cell bodies of neurosecretory cells in the ventral nerve cord (VNC) are mainly found in abdominal neuromeres (A1-A9) and only a set of FMRFamide-expressing cells (Tv) are known in the thoracic neuromeres (T1-T3). **a** Peptide hormones in abdominal neuromeres that regulate water and ion balance, as well as stress responses. The thoracic Va neurons have axon terminations in a neurohemal area in the dorsal neural sheath of the VNC, the others terminate along nerve roots and/or on muscles in the body wall. Peptide acronyms: DH31 and DH44, diuretic hormone 31 and 44; LK, leucokinin; GPB5, glycoprotein B5. **b** Peptides with unclear functions in the adult, except FMRFa neurons (see text). The Tv neurons have axon terminations in a plexus forming a neurohemal area in the dorsal neural sheath of the VNC (see Fig. 2), the others terminate on muscles in the body wall. Additional efferent neurons of abdominal neuromeres (cells not shown) are listed in the box. Peptide acronyms: OK-A, orcokinin A; Burs, bursicon; AstA, allatostatin-A; ITP, ion transport peptide; RYa, RYamide; PDF, pigment-dispersing factor

Peptidergic neurosecretory cells were identified not only in the brain groups (LNCs, MNCs and SNCs), but also in the ventral nerve cord (VNC). Also here, some studies were initially performed with heterologous antisera to NPHs from vertebrates or mollusks. Earlier studies had revealed so-called perivisceral organs (PVOs; also known as parasympathetic organs), which were neurohemal release sites of presumed peptide hormones produced in NSCs of the VNC [reviewed in (Nässel 1996; Predel 2001; Raabe 1989)]. The identities of some of these NPHs were revealed by peptide immunohistochemistry as outlined below. Other peptides were actually isolated from PVOs and subsequently sequenced, for instance periviscerokinin (PVK) (Predel et al. 1995). Later on, also mass spectrometry was used for peptide identification in these organs (Neupert and Predel 2005; Predel 2001; Predel et al. 2003, 2004). Interestingly, adult flies such as *Calliphora* and *Drosophila* do not possess such PVOs. Instead, the axon terminations of peptidergic neurosecretory cells of the VNC are spread out in a plexus in the dorsal neural sheath of the VNC (Lundquist and Nässel 1990; Nässel et al. 1988a) (Fig. 2). Typically, early studies revealed that the thoracic neuromeres of the VNC house NSCs expressing extended FMRFamides (Lundquist and Nässel 1990; Predel et al. 2004; Schneider et al. 1993) and abdominal ones CAPA-Pyrokinin/periviscerokinin, PDF, and LK (Cantera and Nässel 1992; Chen et al. 1994; Nässel et al. 1993; Predel et al. 2004) [see Fig. 3].

Already in the early 1980s, the presence of NPHs in intestinal EECs was discovered. Again these earlier screens were made with heterologous antisera to mammalian peptides, like for instance bovine pancreatic polypeptide (Duve and Thorpe 1982; Endo et al. 1982; Endo and Nishiitsutsuji-Uwo 1981; Sehnal and Žitňan 1990; Žitňan et al. 1993a). A few endogenous insect peptides were, however, identified in EECs in the 1990s, *Manduca* diuretic hormone (Veenstra et al. 1995), allatostatin-A (Yoon and Stay 1995), and TK (Lundquist et al. 1994). Today 10 different NPHs in are known the *Drosophila* gut (Supplemental Fig. 1) (Lemaitre and Miguel-Aliaga 2013; Veenstra et al. 2008).

In mammals, it was known that many peptidergic neurons co-express small molecule neurotransmitters (SMNs) or other neuropeptides (Hökfelt et al. 1986, 1987). Thus, some early attempts were made to discover colocalization in insects, but few were revealed at the time. These include the previously mentioned expression of glutamate and proctolin in motoneurons (Adams and O'Shea 1983; Bishop et al. 1984); several NPHs (allatotropin, allatostatin-A, FMRFamide-like, and tachykinin) were found colocalized with GABA in locust interneurons [summarized in (Homberg 2002; Nässel and Homberg 2006)] and furthermore serotonin/allatostatin-A (Vitzthum et al. 1996), serotonin/PDF (Petri et al. 1995), and octopamine/tachykinin (Vitzthum and Homberg 1998) in other interneurons.

Only a few insect receptors of NPHs were known in the 1990s. These were *Drosophila* G-protein-coupled receptors (GPCRs) with sequence similarities to tachykinin receptors (TkR99D and TkR86C) (Li et al. 1991; Monnier et al. 1992) and to a mammalian NPY receptor (Li et al. 1992). Later a *Drosophila* allatostatin-A receptor (Birgul et al. 1999) and a *Manduca sexta* diuretic hormone receptor (Reagan et al. 1993) were identified. The cellular localization of these receptors was not revealed until many years later (in *Drosophila*), as discussed later.

Finally, there were a few early studies showing electron microscopic immune-localization of neuropeptides using pre- or post-embedding technique (Cantera and Nässel 1991, 1992; Eckert et al. 1981; Nässel et al. 1988a, 1988b, 1995). The former employed peroxidase detection, the latter used so-called immuno-gold labeling, which employed secondary antisera tagged with small gold particles. Axon terminations or axon varicosities were shown to contain large dense core vesicles, often co-localized with smaller clear vesicles.

### Assays of functions of NPHs in early studies

In the early years described above, *Drosophila* was not especially popular for NPH discovery or functional analysis. The small size of *Drosophila* made biochemical identification of peptides difficult with methods available and prior to the development of genetic tools, experimental work was hard. Thus, the work described below focused on larger insects like for instance moths, locusts, cockroaches and the kissing bug *Rhodnius prolixus*.

As mentioned, many NPHs had originally been isolated by classical endocrinology technique where tissue extract was assayed during purification for specific hormonal actions in vivo. Thus, some functions were known at the outset. For instance, AKH was isolated by purification of corpora cardiaca extract that was throughout tested for its lipid-mobilization activity in locusts (Stone et al. 1976). Similarly, the other neuropeptide hormones listed in Table 1 (bombyxin, allatostatins, eclosion hormone, PBAN, and PTTH) had at least one known hormonal function. Therefore, these hormonal peptides were among the NPHs that received further early attention. AKH was subjected to intensive investigation early on and its modes of action in carbohydrate and lipid metabolism determined in numerous studies [see reviews by (Gäde and Auerswald 2003; Gäde et al. 1997; Van der Horst et al. 2001)]. Likewise, the first diuretic hormone (DH) was isolated from the moth *Manduca* by assaying the effect on diuresis (Kataoka et al. 1989b), whereas later some other DHs were discovered by other means (Coast et al. 2002). The allatostatins were isolated by their action to inhibit biosynthesis of juvenile hormone (Kramer et al. 1991; Pratt et al. 1989; Stay and Tobe 2007) but were also found to be myoactive (Lange et al. 1995). Hormonal roles of eclosion hormone and PTTH were also known from original assays (Kataoka et al. 1991, 1987), and their function in development has been intensely studied over the years following their identification [see reviews (Ewer 2005; Marchal et al. 2010; Yamanaka et al. 2013)].

Other neuropeptides, like proctolin, LK, TK, and FMRF-related peptides that had been isolated based on their myotropic activity, were employed extensively in tests of activity on different visceral muscle (Holman et al. 1991, 1990; Schoofs et al. 1993). First of all, monitoring peptide actions on muscle contractions was a simple but powerful assay for discovering new neuropeptides in tissue extracts. Secondly, it was also a convenient in vitro assay for analysis of peptide structure–activity properties. Thus, peptides were synthesized in different modified forms, including “alanine-substitution scans,” to determine essential amino acids and the active core sequences of peptides [see for example (Nachman et al. 1988, 1993)]. Commonly, the carboxy terminus was found essential for activity and often required an alpha-amidation. In some cases, the active cores were found to be as small as pentapeptides, like in cockroach myosuppressin and pyrokinin (Nachman et al. 1993, 1991).

Analysis of AKH peptides initially focused on lipid metabolism, but also carbohydrate regulation and their role in energy allocation during flight [see (Gäde and Auerswald 2003; Gäde et al. 1997; Orchard 1987; Van der Horst 2003; Van der Horst et al. 2001)]. In contrast to most of the other NPHs isolated at the time, the amino-terminus (N-term; commonly pyroglutamate-blocked) was found critical for activity in AKHs. As we shall see below, research on AKH signaling expanded in the “postgenomic era,” with investigations also of the roles in activity, feeding, and energy homeostasis in *Drosophila* in tandem with insulin-like peptides (see Table 2).

**Table 2:** Diverse functions of NPHs produced by brain neurosecretory cells both as hormones and when produced also by other neurons (see Fig. 5)

Function^1^	Neuropeptide	Circuit/neurons^2^	References
Olfaction	DILPs	OSN-PN (AL) OSN (AL)	(Root et al. 2011) (Lebreton et al. 2015)
	Hugin-PK	SEZ-NSC via SIFa neurons to OSNs (AL)	(Martelli et al. 2017)
	sNPF	OSN-PN (AL)	(Root et al. 2011)
	TK	LN-OSN-PN (AL)	(French et al. 2021; Ignell et al. 2009; Ko et al. 2015)
Taste	DSK	MP1/MP3-Gr64f	(Guo et al. 2021)
	Hugin	Hugin neurons (SEZ, larvae)	(Hückesfeld et al. 2016; Melcher and Pankratz 2005; Schlegel et al. 2016)
	LK	SELK neurons (SEZ)	(Chu et al. 2024; Lopez-Arias et al. 2011; Mollá-Albaladejo et al. 2024)
	sNPF	LNCs-Gr66a	(Inagaki et al. 2014; Kim et al. 2013)
	TK	Pheromone pathway	(Shankar et al. 2015)
		Central complex and Gr43a (fructose)	(Musso et al. 2021)
Food search/feeding	CAPA	NSCs brain, VNC	(Koyama et al. 2021)
	CRZ	LNCs	(Kubrak et al. 2016)
	DH31	EECs	(Lin et al. 2022)
	DH44	MNCs and VNC neurons	(Dhakal et al. 2022; Dus et al. 2015; Kasturacharya et al. 2023; Oh et al. 2021; Yang et al. 2018; Zandawala et al. 2018a)
	DILPs	IPCs	(Barber Annika et al. 2021; González Segarra et al. 2023; Li et al. 2024b; Nässel and Vanden Broeck 2016; Owusu-Ansah and Perrimon 2014; Wang et al. 2020)
	DMS	MNCs	(Hadjieconomou et al. 2020)
		Brain neurons	(Wu et al. 2024)
	DSK	IPCs, MP1/MP3	(Guo et al. 2021; Li et al. 2024a; Nässel and Wu 2022; Söderberg et al. 2012)
	Hugin	SEZ neurons (Larvae)	(King et al. 2017; Melcher and Pankratz 2005; Schlegel et al. 2016)
	ITP	LNCs	(Galikova et al. 2018; Gera et al. 2024)
	LK	Brain neurons	(Al-Anzi et al. 2010; Zandawala et al. 2018a)
	sNPF	OSNs-PNs (AL), Local brain interneurons, MB circuits	(de Tredern et al. 2024; Hong et al. 2012; Lee et al. 2004; Root et al. 2011; Shen and Cai 2001; Tsao et al. 2018)
	TK	OSNs-PNs (AL)	(Ko et al. 2015)
		SMP-TK	(Qi et al. 2021)
		Central complex	(Musso et al. 2021)
Thirst/drinking	DILPs	IPCs	(González Segarra et al. 2023)
	LK	Brain neurons	(Chu et al. 2024)
	ITP	LNCs	(Galikova et al. 2018)
Locomotor activity (independent of clock)	DH44	Brain neurons	(Jiang et al. 2024; Zhao et al. 2024)
	DSK	Larvae	(Chen et al. 2012)
	Hugin-PK	Larvae	(King et al. 2017; Schlegel et al. 2016)
	LK	Larvae	(Okusawa et al. 2014)
	TK	Brain neurons	(Zhao et al. 2024)
Clock function/circadian activity/sleep	DH31	Clock circuit	(Goda et al. 2016; Kunst et al. 2014)
	ITP	Clock circuit	(Hermann-Luibl et al. 2014; Le et al. 2024)
	sNPF	Clock circuit	(Liang et al. 2017; Shang et al. 2013)
Activity/sleep	Corazonin	Brain neurons	(Afonso et al. 2015)
	DH31	Central body	(Lyu et al. 2023)
		MNCs	(Chong et al. 2024)
	DH44	MNCs	(Barber et al. 2016; Cavanaugh et al. 2014; Chong et al. 2024; King et al. 2017; Poe et al. 2024)
	DILPs	IPCs	(Barber et al. 2016; Yamaguchi et al. 2022)
	Hugin-PK	SEZ neurons	(King et al. 2017; Schwarz et al. 2021)
	LK	Lateral horn neurons (LHLKs)	(Cavey et al. 2016; Murakami et al. 2016; Murphy et al. 2016; Yurgel et al. 2019)
	sNPF	MB neurons	(Chen et al. 2013)
	TK	Fan-shaped body	(Lee and Kim 2021)
		Antennal lobe	(French et al. 2021)
		Brain interneurons	(Lee et al. 2021)
Aggression^3^	DH44	DH44-R1 cells	(Kim et al. 2018)
	DSK	IPCs	(Agrawal et al. 2020; Luo et al. 2014; Williams et al. 2014)
		MP1/MP3 Brain	(Wu et al. 2020)
	TK	LPP1B brain interneurons	(Asahina et al. 2014; Wohl et al. 2023)
Learning	Corazonin	VNC neurons^4^	(Zer-Krispil et al. 2018)
	DH31	Clock neurons	(Frantzmann et al. 2023)
		OA neurons	(Lyu et al. 2023)
	DILPs	IPCs	(Chambers et al. 2015; Naganos et al. 2012; Tanabe et al. 2017)
	LK	Brain neurons	(Senapati et al. 2019)
	sNPF	Mushroom Body	(Knapek et al. 2013)
Nociception	Corazonin	Brain neurons	(Nakamizo-Dojo et al. 2023)
	DSK	Descending neurons; Larvae	(Oikawa et al. 2023)
	LK	LK receptor	(Ohashi and Sakai 2018)
		LK neurons (VNC in larvae)	(Imambocus et al. 2022; Li et al. 2023)
	sNPF (DILP7)	Interneurons (VNC in larvae)	(Hu et al. 2017)
	TK	VNC (larvae)	(Gu et al. 2022; Im et al. 2015)
		Brain neurons (adult); visual aversion	(Tsuji et al. 2023)
Courtship and reproduction	Corazonin	VNC neurons	(Tayler et al. 2012; Zer-Krispil et al. 2018)
		Brain neurons	(Bergland et al. 2012; Bonheur et al. 2023; Lin et al. 2022; Zhang et al. 2024)
	DH44	MNCs	(Lee et al. 2015)
		PC1 neurons	(Jiang et al. 2024; Kim et al. 2024; Zhao et al. 2024)
	DILPs	IPCs	(Chen et al. 2022; Lebreton et al. 2015; Zhang et al. 2022)
	DSK	MP1/MP3	(Li et al. 2024a; Wu et al. 2019)
		IPCs	(Fedina et al. 2023)
	LK	VNC neurons	(Liu et al. 2020)

This table lists both hormonal functions (for brain NSCs) and functions when the same peptides are expressed by other neurons.

^1^Some functions listed are indirect and some are classified very generally.

^2^Some neuron types are listed by acronyms (see list below).

^3^In males if not indicated.

^4^A successful copulation is a reward in male flies and strengthens long-term appetitive memories. Corazonin signaling induces ejaculation, which in turn affects NPF signaling and reward/memory.

*Peptide acronyms*: see list of abbreviations

Other acronyms: *AL* antennal lobe, *Gr64f, Gr66a* gustatory receptors, *DH44-R1* neurons expressing DH44 receptor, *IPCs* insulin-producing cells, *LHLKs* leucokinin neurons in lateral horn of brain, *LN* local neurons, *LNCs* lateral neurosecretory cells, *MB* mushroom bodies, *MNCs* median neurosecretory cells, *MP1/MP3* DSK expressing interneurons, *LPP1B* and *SMPTK* subset of TK neurons, *OA* octopamine, *OSN* olfactory sensory neurons, *OS-PN* olfactory lobe projection neurons, *PC1 neurons* set of neurons regulating sex-specific behavior, *PN* projection neurons, *SEZ* subesophageal zone, *SMP-TK* TK expressing interneurons, *VM2* pheromone-sensitive glomerulus in antennal lobe

The first insect diuretic hormone to be fully sequenced was the corticotropin-releasing factor (CRF)-like diuretic hormone (DH41) discovered in *Manduca* in bioassays for diuretic activity (Kataoka et al. 1989b). Other already sequenced NPHs were found diuretic in bioassays ex vivo*:* LK (Coast et al. 1990; Terhzaz et al. 1999), CAP_2B_ (CAPA-PVK) (Davies et al. 1995), the DH41 relative in *Drosophila* (DH44) (Cabrero et al. 2002), and a calcitonin-like diuretic peptide (DH31) (Coast et al. 2001; Furuya et al. 2000). Also, a putative antidiuretic hormone, ion transport peptide (ITP) was identified in a locust (Meredith et al. 1996; Phillips et al. 1998). It can be noted that an early study identified a vasopressin-like peptide (VPL) in locusts and demonstrated its diuretic activity (Proux et al. 1987). However, it was later shown that VPL has no direct activity on renal tubules (Coast et al. 1993). Many years later, studies in the red flour beetle, *Tribolium castaneum*, suggested that VPL (now designated inotocin) is produced by interneurons and acts via other neurons to regulate diuresis (Aikins et al. 2008). Also this area of NPH signaling grew and is still very active, now dealing with a large number of diuretic and anti-diuretic peptides, their receptors, and downstream signaling mechanisms, as well as mechanisms of action in epithelia. As we shall see in a later section, the diuretic hormones have been found more recently to be highly pleiotropic and regulate additional phenomena as disparate as feeding and stress, and several are used by neurons of the circadian clock in *Drosophila* [see (Nässel and Zandawala 2019; Reinhard et al. 2024)].

In the mid-1990s, basically nothing was known about roles of insect neuropeptides in brain circuits (Homberg 2002; Nässel 1993b). Only speculations about functions existed that were based on the distribution of peptidergic neuronal processes in different regions of the brain. For instance, the presence of a neuropeptide in the antennal lobe suggested a role in processing of olfactory information [see (Nässel 1993b)]. The discovery of *Drosophila* PDF in certain clock neurons (characterized by expression of the clock gene *period*) (Helfrich-Förster 1995) suggested the role of this peptide as a major signal in circadian clock function. A few years later a landmark paper used genetic tools to confirm PDF as functional clock peptide (Renn et al. 1999). The roles of PDF and other NPHs in the clock circuitry and sleep regulation in *Drosophila* and other insects became an intensively investigated area that is still very active today [see (Beer and Helfrich-Förster 2020; Muraro et al. 2013; Nitabach and Taghert 2008; Reinhard et al. 2024)].

### NPHs enter the genomic and postgenomic era

The sequencing and annotation of the *C. elegans* and *D. melanogaster* genomes (Adams et al. 2000; Bargmann 1998; Sequencing Consortium 1998) meant that a multitude of genes associated with NPH precursors and receptors were identified. This enabled the association of all (or most of) the biochemically identified NPHs with precursors, and one thereby obtained a first handle on the total number of NPHs in an organism. Also, annotations of *Drosophila* GPCRs followed [see (Hewes and Taghert 2001; Vanden Broeck 2001a)], prompting efforts to identify their NPH ligands. When further genomes were sequenced, it was possible to establish relations between GPCRs, NPHs (and their precursors) in different species and obtain clues to the evolutionary relations of these signaling molecules among metazoans (Elphick et al. 2018; Jékely 2013; Mirabeau and Joly 2013). As already suggested before the genome sequencing [see (De Loof and Schoofs 1990)], we know today that the structures of a number of NPHs and their GPCRs are fairly well conserved over bilaterian evolution (Jékely 2013; Mirabeau and Joly 2013) whereas others appear to be taxon-specific. These recent papers show that about 25 NPH signaling systems in insects (NPHs and cognate GPCRs) can be found also in vertebrates.

Importantly, with the orphan GPCRs (with unknown ligands), a large number of novel NPHs were discovered that warranted characterization. This opened an active explorative era in insect NPH research and numerous publications described annotation and characterization of novel NPHs and GPCRs [see (Caers et al. 2012; Hauser et al. 2006; Hewes and Taghert 2001; Hill et al. 2002; Liu et al. 2006; Vanden Broeck 2001a, 2001b)], and efforts are still made to extend the number of complete insect peptidomes with new species.

Along with the discovery of almost complete sets of NPHs and GPCRs, exploration of their function was initiated in the early 2000s using novel technology. With the introduction of the binary GAL4-UAS system in *Drosophila* (Brand and Perrimon 1993), it became possible to target fluorescent markers (GFP) to visualize specific sets of peptidergic neurons, to ablate them by means of apoptosis genes, to manipulate their membrane activity, or to diminish the expression of specific genes, including those that encoded NPH precursors or GPCRs [see (Duffy 2002; Guo et al. 2019)]. Two early papers pioneered this kind of study in *Drosophila*; one demonstrated the role of PDF in specific clock neurons (Renn et al. 1999), and the other the role of eclosion hormone in ecdysis (McNabb et al. 1997). By now, the number of studies using GAL4-UAS and LexA-LexAop systems and their variants in NPH research is far too numerous to cite here. These binary systems are now possible to induce conditionally (temporally) for instance by using Gal80 or Gene switch technique, and spatially in restricted sets of neurons by intersectional techniques [see (Duffy 2002; Guo et al. 2019)]. Thus, it is possible to target single neurons at a desired time-point in the life cycle (or time of day). Huge libraries of GAL4 and LexA and split-GAL4 driver lines are publicly available (e.g., FlyLight, https://flweb.janelia.org/cgi-bin/flew.cgi) (Meissner et al. 2023; Pfeiffer et al. 2008). Further *Drosophila* resources are available via FlyBase (http://flybase.org/). Another major advance was techniques for imaging of calcium activity in neurons by expression of genetically encoded Ca^2+^ sensors with the GAL4-UAS system [see (Simpson and Looger 2018)]. It is now possible to genetically express a thermo- or chemo-sensitive channel in specific neurons (to activate them) and express Ca^2+^ sensors in their putative target neurons and thereby confirm connectivity [see (Guo et al. 2019)]. Synaptic connectivity can furthermore be studied by trans-Tango or GRASP technique and variants thereof (Guo et al. 2019; Shearin et al. 2018; Talay et al. 2017; Timalsina et al. 2024), with chemoconnectomics tools (Deng et al. 2019), and also by electron microscopy of serial sections to assemble a complete synaptic connectome of the *Drosophila* brain [see (Dorkenwald et al. 2024)]. The brain connectome is an invaluable resource for revealing functional neuronal circuits; however, it may be less useful for analysis of peptidergic signaling. A problem is that NPHs appear to signal predominantly as non-synaptic messengers (paracrine signaling) and thus largely independent of synaptic contacts [see (Bargmann 2012; Bargmann and Marder 2013; Marder 2012; Nässel 2009; Nusbaum et al. 2017; Salio et al. 2006)]. One way to overcome this problem is to combine synaptic connectome analysis with single-cell transcriptome data of GPCR expression. This way it may be possible to identify neuronal targets of nearby peptidergic “sender neurons” as shown for instance in the *Drosophila* clock circuit and the networks formed by NSCs in the same species (McKim et al. 2024; Reinhard et al. 2024; Schlegel et al. 2016). In summary so far, we are in a favorable situation where analysis of NPH function in *Drosophila* is amenable to detailed analysis. So what has been accomplished with these novel techniques over the last few years? Due to space limitations, it is not possible to assemble a comprehensive summary of all the findings available on insect NPHs. Instead, I selected some examples (see also Table 2) that illustrate the complexity in NPH signaling in the humble fly *Drosophila* that may be generally valid also for other animals.

### A brief overview of NPH signaling in *Drosophila*

**Fig. 4:**
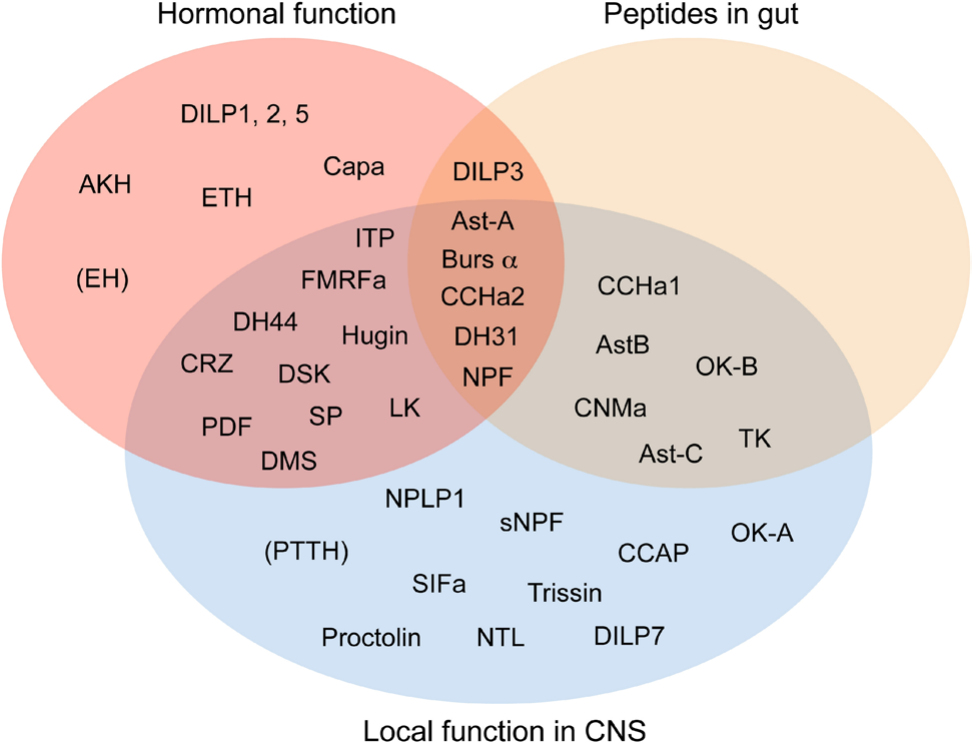
Peptide distribution patterns in *Drosophila*. This Venn diagram displays patterns of distribution of NPHs in hormonal systems, brain interneurons and the intestine. Note that many peptides are part of all three systems, some in two, and relatively few are only hormonal or interneuronal. There are so far no neuropeptides known to be unique to the intestine. Peptides in brackets have not been demonstrated in mature adult structures. The peptide acronyms are listed separately. Note that some *Drosophila* peptides are missing in this figure due to lack of clear information. This figure is updated from (Nässel et al. 2019)

About 50 NPH precursors and receptors had been identified in *Drosophila* in 2019 (Nässel and Zandawala 2019). A few *Drosophila* NPHs have been added since 2019, marmite (Francisco et al. 2022), nesfatin-1 (Yañez-Guerra et al. 2022), sparkly (Sukumar et al. 2024), and phoenixin (Zandawala et al. 2024); these peptides and a few others have not yet been paired with receptors. In *Drosophila*, most NPHs each act on a single receptor, but for 10 NPHs two GPCRs have been identified [see (Nässel and Zandawala 2019)]. Conversely, it has been found that several of the eight *Drosophila* insulin-like peptides converge on a single receptor (dInR, a receptor tyrosine kinase) (Brogiolo et al. 2001; Nässel and Vanden Broeck 2016), whereas two (ILP7 and 8) act on GPCRs (Colombani et al. 2015; Garelli et al. 2015; Imambocus et al. 2022). As mentioned, the cellular distribution of NPHs varies with some being produced by only a few neurons and others in larger numbers of neurons, NSCs and gut EECs. 40 of these NPHs are shown in Fig. 4 divided up functionally into roles as hormones, presence in gut EECs and being utilized by local interneurons. It can be seen that most of the NPHs have at least two such functions, and some have three. As an example, DH31 is expressed in gut EECs, in clock neurons (and other interneurons) as well as in NSCs, whereas SIFamide is only produced by four interneurons with branches throughout the brain. No NPH is expressed only in gut EECs.

**Fig. 5:**
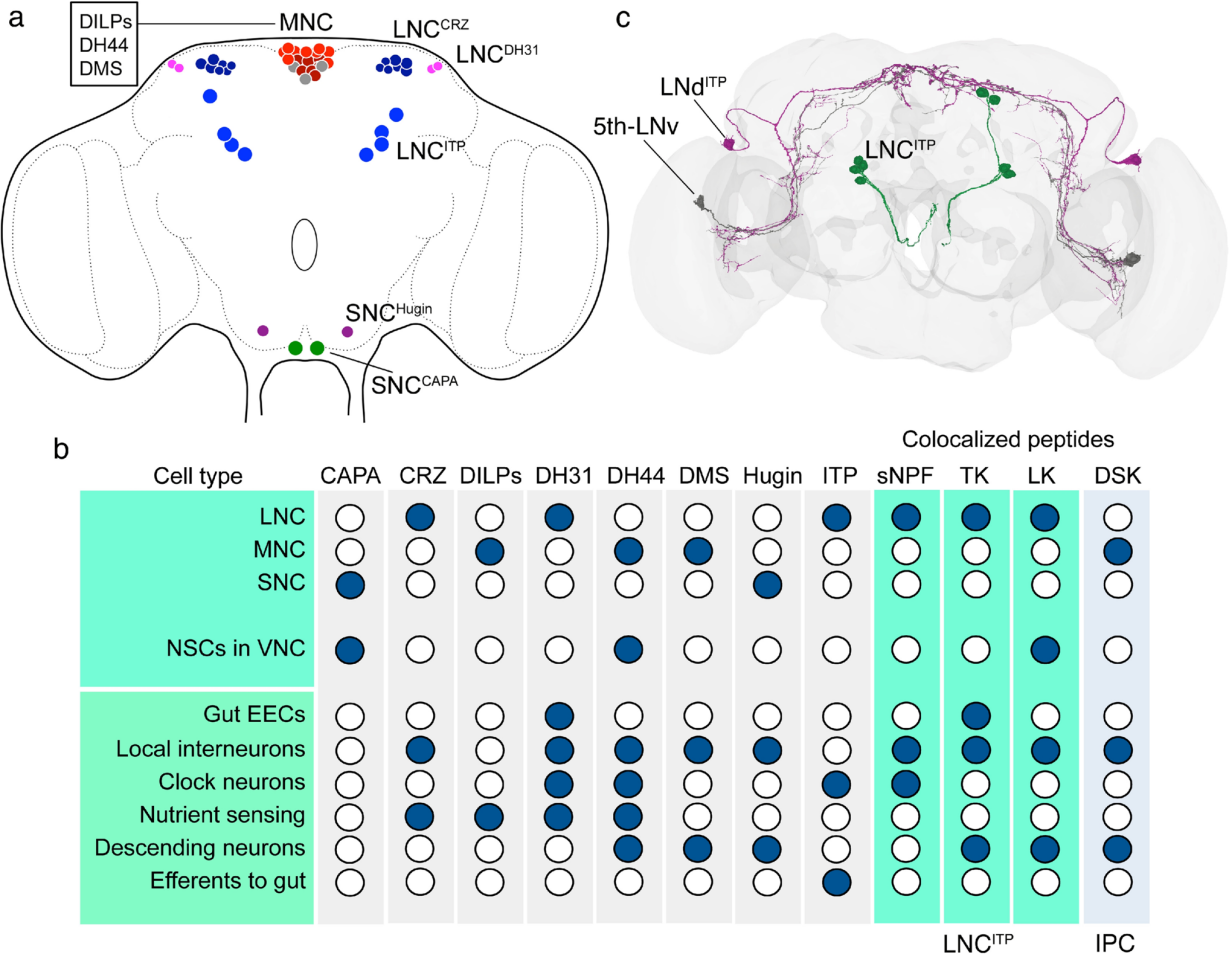
NPHs found in brain neurosecretory cells (NSCs) can also be identified in other types of neurons and endocrine cells. **a** Brain NSCs in *Drosophila*. Median NSCs (MNCs) consist of three main types that either produce insulin-like peptides (DILPs), diuretic hormone 44 (DH44) or myosuppressin (DMS). LNCs produce corazonin (LNC^CRZ^), DH31 (LNC^DH31^) and ion transport peptide (LNC^ITP^). In the subesophageal zone, two types of NSCs are found, SNC^Hugin^ that express hugin pyrokinin and SNC^CAPA^, expressing CAPA peptides. **b** A table showing the distribution of NPHs in the different types of NSCs and in other cells types. Note that four NPHs are included that are co-localized in LNC^ITP^ and in insulin-producing cells (IPCs). These are short neuropeptide F (sNPF), tachykinin (TK), leucokinin (LK), and sulfakinin (DSK). **c** An example of two other neuron types expressing ITP. These are two types of clock neurons (LNd^ITP^ and 5th-LNv). Panel **c** is slightly altered from (Gera et al. 2024)

**Fig. 6:**
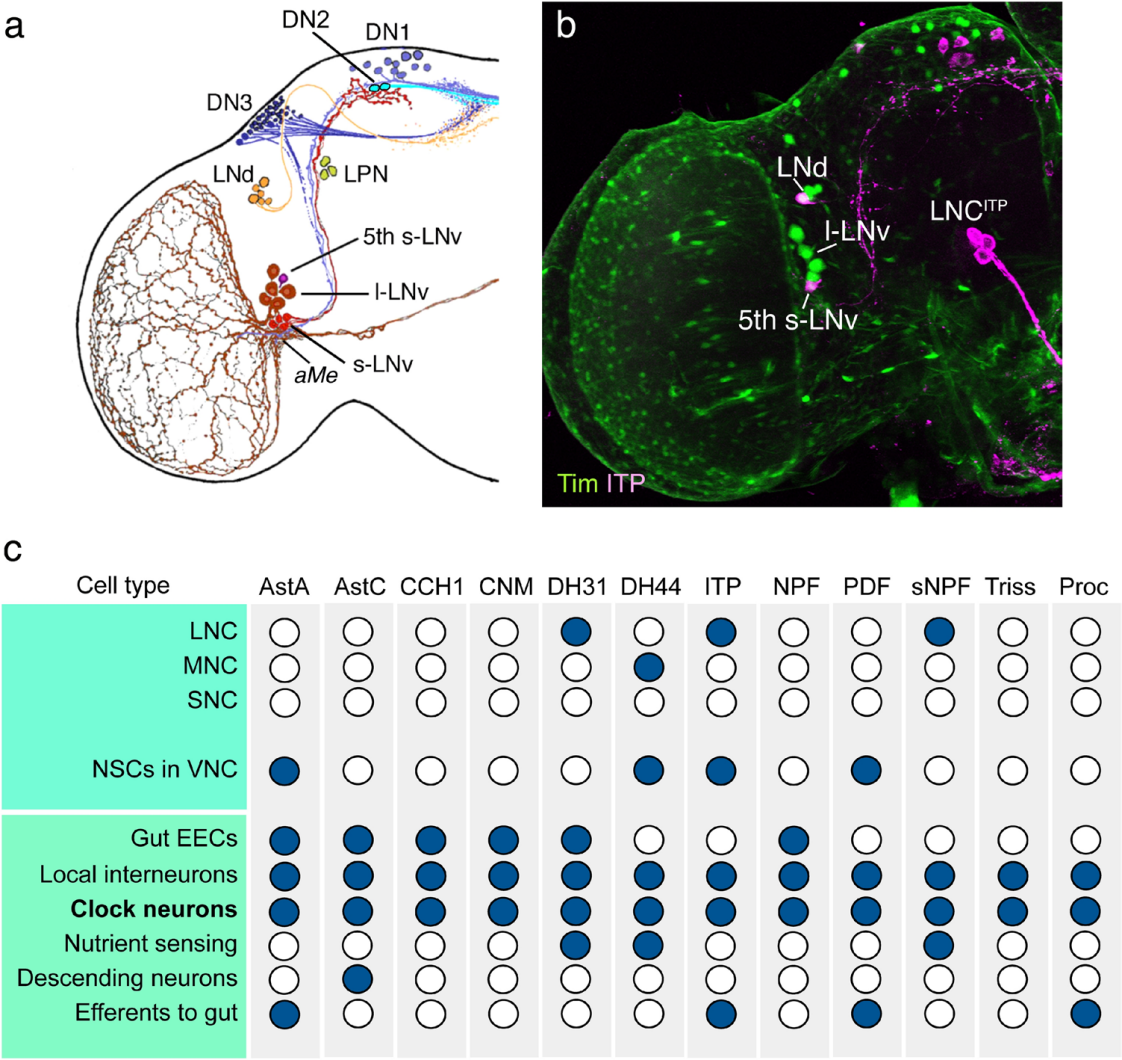
NPHs found in clock neurons of *Drosophila*. **a** Clusters of cock neurons in the left brain hemisphere. There are four main types of lateral neurons (l-LNv, s-LNv, 5th s-LNv LNd) and three clusters of dorsal neurons (DN1–DN3) and a cluster of LPN neurons. **b** Micrograph of some of these neurons shown by expression of the clock gene *timeless* (*Tim*-GAL4, green) and immunolabeling with antiserum to ITP (magenta). **c** Table showing that the peptides found in clock neurons, also can be detected in other neuron types. Note that PDF is localized to a set of efferent neurons in the abdominal neuromeres of the VNC (Nässel et al. 1993) that are likely NSCs acting on renal tubules (Talsma et al. 2012) and ITP is present in putative NSCs in wing and haltere nerves of the VNC (Dircksen et al. 2008). Peptide acronyms: AstA, allatostatin A; AstC, allatostatin C; CCH1, CCHamide-1; CNM, CNMamide; DH31, diuretic hormone 31; DH44, diuretic hormone 44; ITP, ion transport peptide; NPF, neuropeptide F; PDF, pigment dispersing factor; sNPF, short neuropeptide F; Triss, trissin. For references, see Table 2. Panels **a** and **b** are from (Johard et al. 2009) with permission

If we look at NPH distribution in more detail, we find an even greater diversity. For example, the different brain NSCs (Fig. 5a) are known to produce eight primary NPHs (Fig. 5b) (McKim et al. 2024; Nässel and Zandawala 2019). Additionally, some of the insulin-producing cells of the MNC group also express sulfakinin (DSK) (Söderberg et al. 2012), and the LNC^ITP^ co-express sNPF, TK (Kahsai et al. 2010), and LK (de Haro et al. 2010; Zandawala et al. 2018b). This LK expression is seen in the larval LNC^ITP^ cells but is variable in adults. NPHs that are found in NSCs have also been detected in other neurons in the brain, VNC, or gut EECs, in different patterns for each NPH (Fig. 5b). For instance, ITP is expressed in LNCs and two types of clock neurons (Fig. 5c). A similar scheme can be made for NPHs produced by brain clock neurons (Fig. 6). Until recently, about 150 clock neurons were distinguished (see Fig. 6a, b) (Nitabach and Taghert 2008). However, a novel investigation lists 240 clock neurons (Reinhard et al. 2024). Twelve different NPHs have been identified in different distribution patterns in these neurons (Abruzzi et al. 2017; Reinhard et al. 2024). In Fig. 6c, the distribution of these clock NPHs in other cell types is shown: some NPHs are also found in NSCs of brain or VNC, in gut EECs, and all can be found in other types of brain interneurons.

The above schemes are just three examples of the diversity of cellular expression and possible functions of *Drosophila* NPHs; many more could be shown if space allowed. Next, we shall look into the role of NPHs in regulation of physiology and behavior and pleiotropic actions of these messengers.

### The pleiotropic functions of NPHs

A large number of published studies, especially in the last 10 years, have provided data on the numerous functions of insect NPHs in physiology and behavior (see Table 2). It is apparent that each NPH can play multiple functional roles and also that several different NPHs may converge on regulation of common aspects of physiology and/or behavior [or even converge on the same neuron(s)]. Furthermore, NPHs have been found to act at different levels, commonly arranged in a hierarchy: (1) local neuromodulation in specific circuits, (2) integrating several circuits and/or modalities, and (3) in global, state-dependent, orchestration of behavior and physiology (Anderson 2016; Kim et al. 2017; Nässel et al. 2019; Nässel and Zandawala 2022; Orchard and Lange 2024; Schoofs et al. 2017). One can also classify NPH signaling based on “network interactions,” as proposed in *C. elegans* NPH signaling (Ripoll-Sánchez et al. 2023; Watteyne et al. 2024). These authors divide NPH networks into (1) local (few functionally interconnected neurons), (2) broadcasting (few sender neurons and many target neurons), (3) integrative (few neurons receiving inputs from many neurons), and (4) pervasive (highly interconnected networks with both integrative and broadcasting functions). Interconnected here means pairs of neurons, one expressing a NPH and the other its GPCR (since *bona fide* synapses probably can be disregarded). It is of note that NPH signal networks may be further elaborated (with increased functional flexibility) by existence of more than one GPCR for a given NPH and by neurons co-expressing multiple NPHs and small molecule neurotransmitters. The roles of NPHs in interorgan signaling constitute another addition to our understanding of complex hormonal actions to maintain systemic homeostasis (Nässel and Zandawala 2020, 2022; Okamoto and Watanabe 2022; Owusu-Ansah and Perrimon 2014; Rajan and Perrimon 2011). This section will highlight some of these phenomena.

**Fig. 7:**
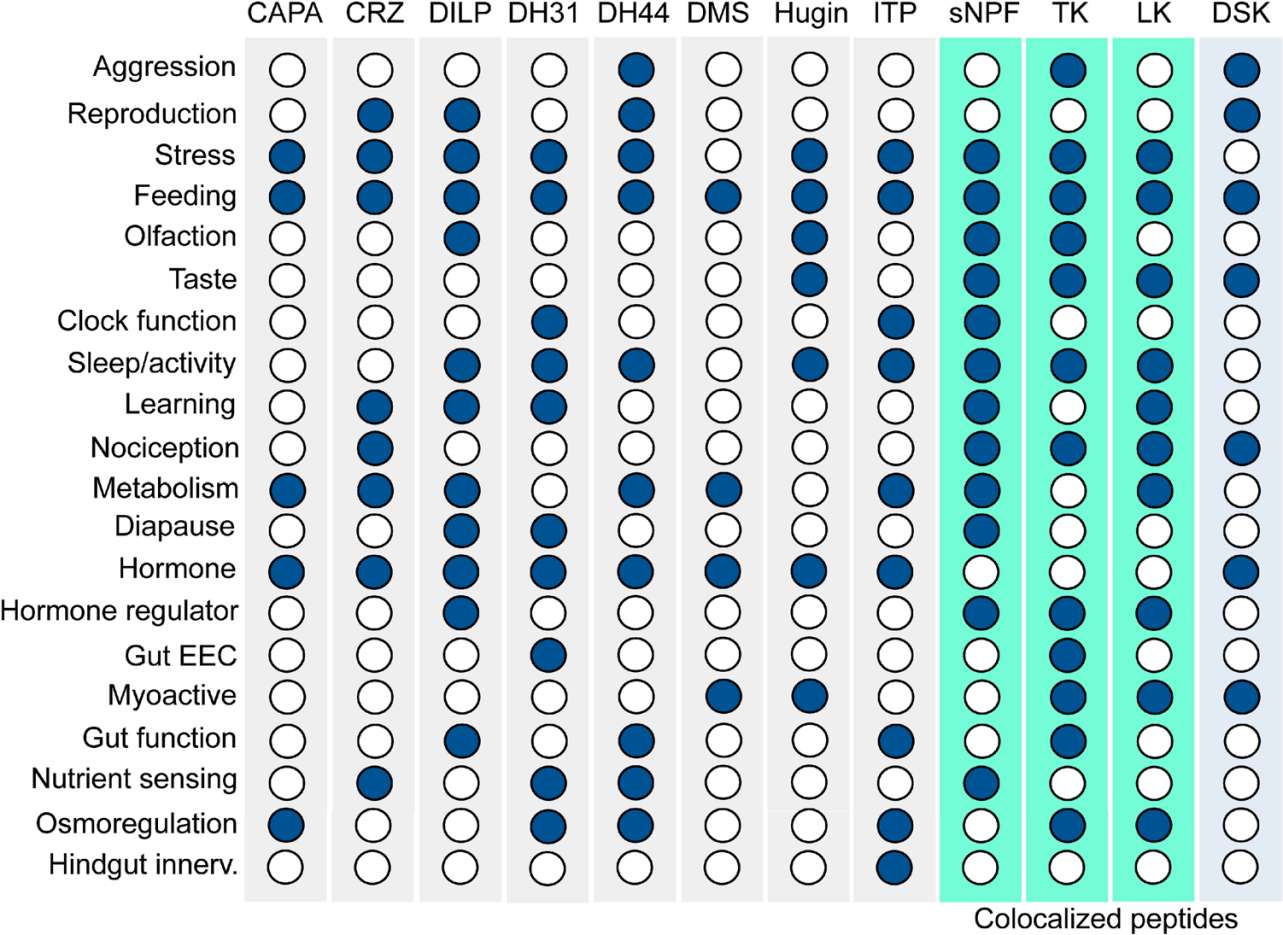
Table showing the different functions of NPHs produced by NSCs. NPH acronyms are as in Fig. 5. Note that some of the functions provided need some further definition. For example, reproduction is primarily reproductive behavior (not development or maturation of ovaries or eggs); stress includes responses to cold, as well as osmotic and metabolic stress; feeding includes hunger, food search, and food ingestion; sleep/activity includes activity rhythm and *bona fide* sleep, whereas clock function indicates that the NPH is acting with clock neuron circuit; nutrient sensing is in either peptidergic neurons of the brain or in EECs of the gut. For references, see Table 2

When the distribution of NPHs had been mapped in *Drosophila* as outlined above, it was obvious that some NPHs could act both as neuromodulators in different neuronal circuits (locally or more globally), in efferent neurons to target muscles or other peripheral tissues, and as circulating hormones released from neurosecretory cells (NSCs) or endocrine cells. Thus, one basis for this multiplicity is the expression of a given neuropeptide in different types of neurons (and circuits), NSCs and EECs. Indeed, as outlined in Fig. 7 and Table 2, experimental work has confirmed that many peptides have multiple roles in the CNS and at peripheral targets. Since so much data are available, I have focused on a subset of the known NPHs. Thus, I limit myself here to the 8 *Drosophila* NPHs that are produced by brain NSCs (in the three different groups) and shown to act as hormones, and four other NPHs that are co-localized in two of the NSC types (Fig. 7). As seen in Fig. 7, the number and types of pleiotropic actions vary between NPHs. Hence, DH31 and DH44 have 10 major functions each, whereas CAPA and DMS only have four each. It is not unlikely that the smaller number of functions identified for those peptides is due to lack of investigations, rather than them being less pleiotropic. Shown in Fig. 7 are three NPHs that are co-expressed in a set of LNCs (designated ALKs or LNC^ITP^) that primarily produce ITP (Dircksen et al. 2008): sNPF, TK, and LK, the latter with variable expression in adults (Kahsai et al. 2010; Zandawala et al. 2018b). These are peptides that are also known to have multiple functions (sNPF and TK with 12 functions each) as seen in Fig. 7 and Table 2.

It is furthermore apparent that many different NPHs converge on common targets (neuronal circuits or peripheral targets) to regulate specific behavior or physiology. For instance, as seen in Fig. 7, all 12 NPHs produced by brain NSCs regulate different aspects of feeding (see also Table 2). This includes hunger and satiety signaling, foraging, food ingestion, and signaling from a variety of cells (gut EECs, NSCs in brain and VNC, and different types of interneurons) (Table 2). In addition, NPH modulation of olfaction and taste can influence hunger and feeding behavior [see (Guo et al. 2021; Kim et al. 2017)]. As shown in Table 2, the NPH effects on feeding are due to action by a variety of neuron types utilizing these NPHs in addition to the NSCs.

Thus, for instance, DSK acts primarily via a set of two pairs of widely branching interneurons (MP1/MP3), DH31 via gut EECs, and DH44 by both MNCs and NSCs in the abdominal neuromeres of the VNC. The NPHs co-localized with ITP (sNPF, TK, and LK) are likely to regulate feeding via interneuronal signaling in the brain and not as hormones (but experimental evidence is needed). In Fig. 7, it can furthermore be seen that many of the NPHs regulate various aspects of stress (metabolic, osmotic, and temperature) and nociception, and an overlapping set is involved in locomotor activity and/or sleep. Of the 12 NPHs found in brain NSCs, four (DH31, DH44, ITP, and sNPF) have also been detected in neurons of the clock network and are known to partake in clock function and regulation of activity/sleep (Kunst et al. 2014; Ma et al. 2021; Reinhard et al. 2024). Note that DH31 and sNPF also regulate activity/sleep via release from brain interneurons innervating the central body [DH31 (Lyu et al. 2023)] or mushroom bodies [sNPF (Chen et al. 2013)]. DH44 additionally modulates sleep via brain MNCs (King et al. 2017). The remaining five NPHs that regulate activity/sleep (DILPs, Hugin, LK and TK) do so by release from NSCs or interneurons (see Table 2 and references therein).

A similar analysis can be made for example for the 12 different NPHs produced by clock neurons (Reinhard et al. 2024). These NPHs are also produced by other types of neurons (see Fig. 6) and thus serve additional functions unrelated to the clock circuitry. Due to space limitations, I will not go into further detail about functions of these NPHs, but refer to Table 2 where some functions are listed for the peptides produced in clock neurons shown in Fig. 6. Note also that single-cell transcriptome analysis has detected multiple NPHs in the different cell types of the clock system, leading to the identification of many subtypes of clock neurons (Abruzzi et al. 2017; Reinhard et al. 2024). Thus, we have seen a major increase in the number of clock NPHs since 2009 when only four were known (PDF, sNPF, NPF, and ITP) in this system (Johard et al. 2009) and also obtained a more detailed map of their cellular distribution.

The above examples of pleiotropic actions and convergent functions of many NPHs illustrate the complexity in NPH signaling, and we have seen how NPHs have taken the front seat in studies of the regulation of behavioral switches. Insect NPHs have advanced from myoactive and diuretic molecules or specific developmental hormones to being the most versatile regulators of behavior and physiology. Table 2 lists a number of functional roles that were unheard of in the 1980s, but are intensely investigated today: aggression, courtship and reproduction, nociception, learning, activity/sleep, and food search/feeding. To that list, one could add NPH functions in metabolism, immune responses, and other physiologies, as well as development [see reviews (Nässel and Zandawala 2019, 2020; Schoofs et al. 2017)].

**Fig. 8:**
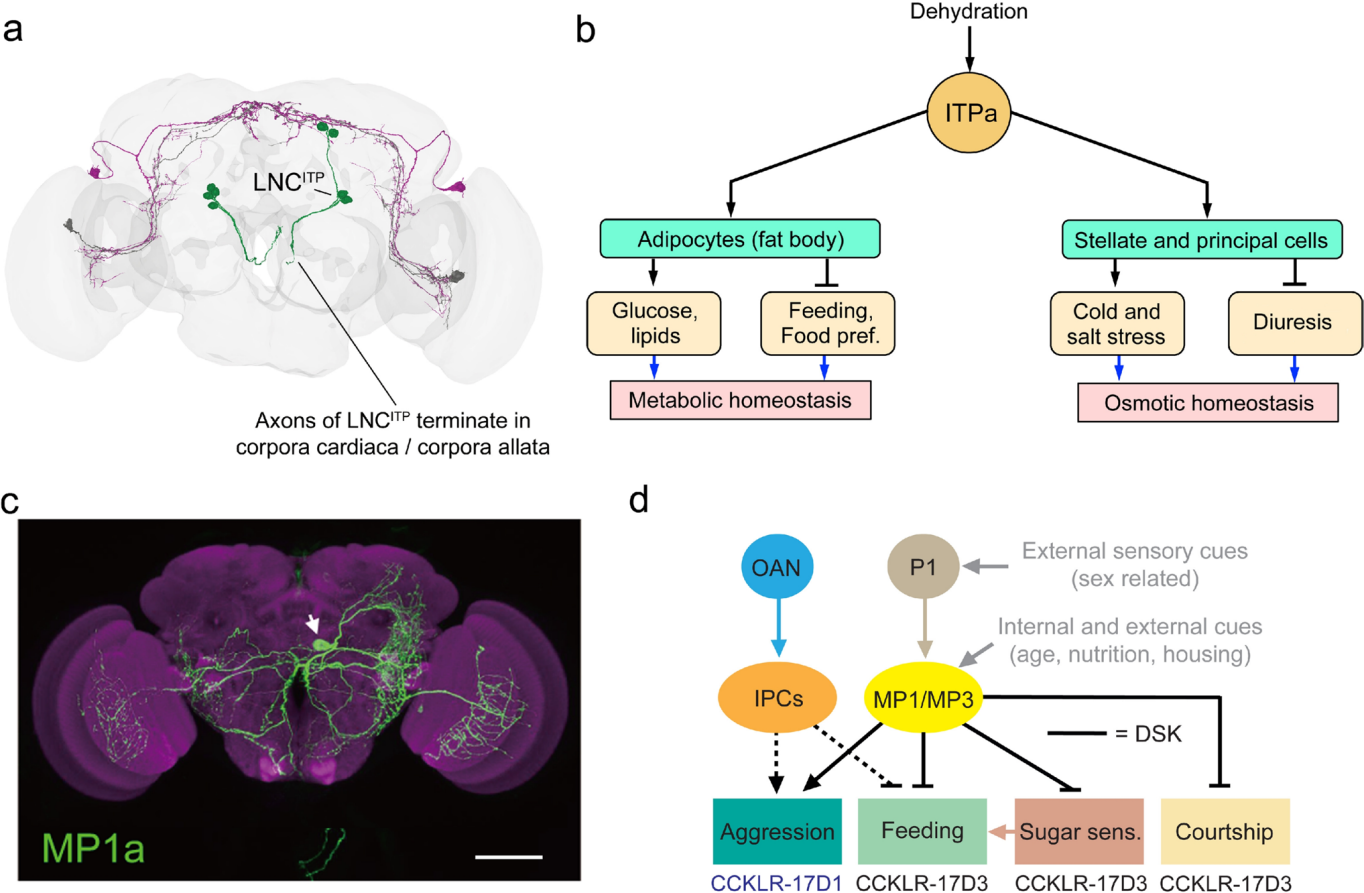
Multiple functional roles of NPHs in *Drosophila* behavior and homeostasis. **a** LSC^ITP^ release ITP into the circulation to affect systemic homeostasis. **b** ITP signaling (ITPa) is triggered by desiccation and the NPH acts via the circulation on adipocytes in fat body and the two main cell types of the renal (Malpighian) tubules. This regulates both metabolic and osmotic homeostasis. **c** A set of six brain interneurons that express sulfakinin (DSK) regulates several behaviors. Here one of the six MP neurons (MP1a) is shown. **d** In male flies, the MP1 and MP3 neurons receive inputs from male-specific P1 neurons (that integrate external sensory input) as well as other input. They act to stimulate aggression and to inhibit feeding and courtship. Part of the feeding inhibition is due to action on sweet taste receptors (sugar sensitivity). Note that two different DSK receptors underlie the antagonistic actions (CCKLR-17D1 and CCKLR-17D3). Also insulin-producing cells (IPCs) act on aggression and feeding [and are regulated by octopaminergic neurons (OANs)]. Panel **a** is slightly altered from (Gera et al. 2024), **b** is summarizing data from (Gera et al. 2024), **c** is from (Guo et al. 2021), and **d** is slightly altered from (Nässel and Zandawala 2022), all with permission

Another interesting aspect of NPH signaling is that a single NPH can serve in neurons to coordinate behavior and physiology/metabolism to obtain a specific outcome (e.g., metabolic and osmotic homeostasis, drinking, or feeding) or to serve as a switch between opposing behaviors (e.g., aggression and courtship). An example of the first is a set of LNCs producing ion transport peptide (ITP) (Fig. 8a). These LNC^ITP^ release ITP that acts on adipocytes to regulate glucose and lipid levels, as well as feeding and food preference, and act on renal tubules to regulate diuresis and cold and osmotic stress responses (Fig. 8b) (Gera et al. 2024). ITP also regulates defecation, probably by action on the hindgut. Taken together, these LNCs use ITP to establish systemic homeostasis in response to dehydration (Gera et al. 2024).

An example of regulation of conflicting behaviors by a small set of neurons involves sulfakinin (DSK) released from four pairs of neurons (MP1 and MP3) in the brain. The MP1a and MP1b neurons arborize widely and bilaterally in the brain (Fig. 8c) and send axons to the VNC, whereas MP3 neurons display more restricted branches unilaterally. Experiments have not clearly distinguished between actions of these different MP neurons, but it was found that in male flies MP1/MP3 neurons stimulate aggression, whereas they inhibit courtship and feeding (Fig. 8d) (Guo et al. 2021; Wu et al. 2020, 2019). Interestingly, DSK acts on two different GPCRs (CCKLR-17D1 and CCKLR-17D3) to mediate the opposing behaviors (Wu et al. 2020, 2019) (Fig. 8d). The MP1/MP3 neurons receive inputs from P1 neurons in the brain, which are a cluster of male-specific neurons known to regulate sex-specific behavior in response to external sensory cues, and the MP1/MP3 neurons also receive signals about nutritional state, age, and housing conditions (Guo et al. 2021; Wu et al. 2020, 2019) (Fig. 8d).

This divergent type of signaling involving two receptor types shown for DSK is understudied in *Drosophila* compared to for instance *C. elegans* (Ripoll-Sánchez et al. 2023; Watteyne et al. 2024). However, there is another *Drosophila* example. This is TK signaling in regulation of aggression where two different GPCRs mediate different levels of aggressive behavior; one receptor (TkR86c) is activated in a local circuit in lower levels of aggression, and the other (TkR99D) is additionally activated in a different circuit at higher levels (Wohl et al. 2023).

A further example of a small set of neurons regulating multiple behaviors is the four brain neurons with extensive branching that produce SIFamide. These neurons receive inputs from multiple brain neurons and modulate activity in circuits regulating taste, olfaction, courtship behavior and activity/sleep, and serve as a hub to modulate appetite, courtship, feeding rhythm, and sleep (Huang et al. 2021; Martelli et al. 2017; Park et al. 2014; Terhzaz et al. 2007).

A final example of an NPH with pleiotropic actions is LK, one of the first NPHs to be isolated back in 1986 in a hindgut assay (Holman et al. 1986a). A single pair of LK expressing neurons (LHLKs) in the brain integrates clock inputs and inputs signaling thirst and hunger, and regulates nutrient-dependent sleep and food choice via action on circuits in the central complex and on insulin-producing cells (Cavey et al. 2016; Sareen et al. 2021; Yurgel et al. 2019). LHLKs furthermore regulate thirst-and sugar-related memory via dopaminergic neurons and the mushroom body (Senapati et al. 2019).

**Fig. 9:**
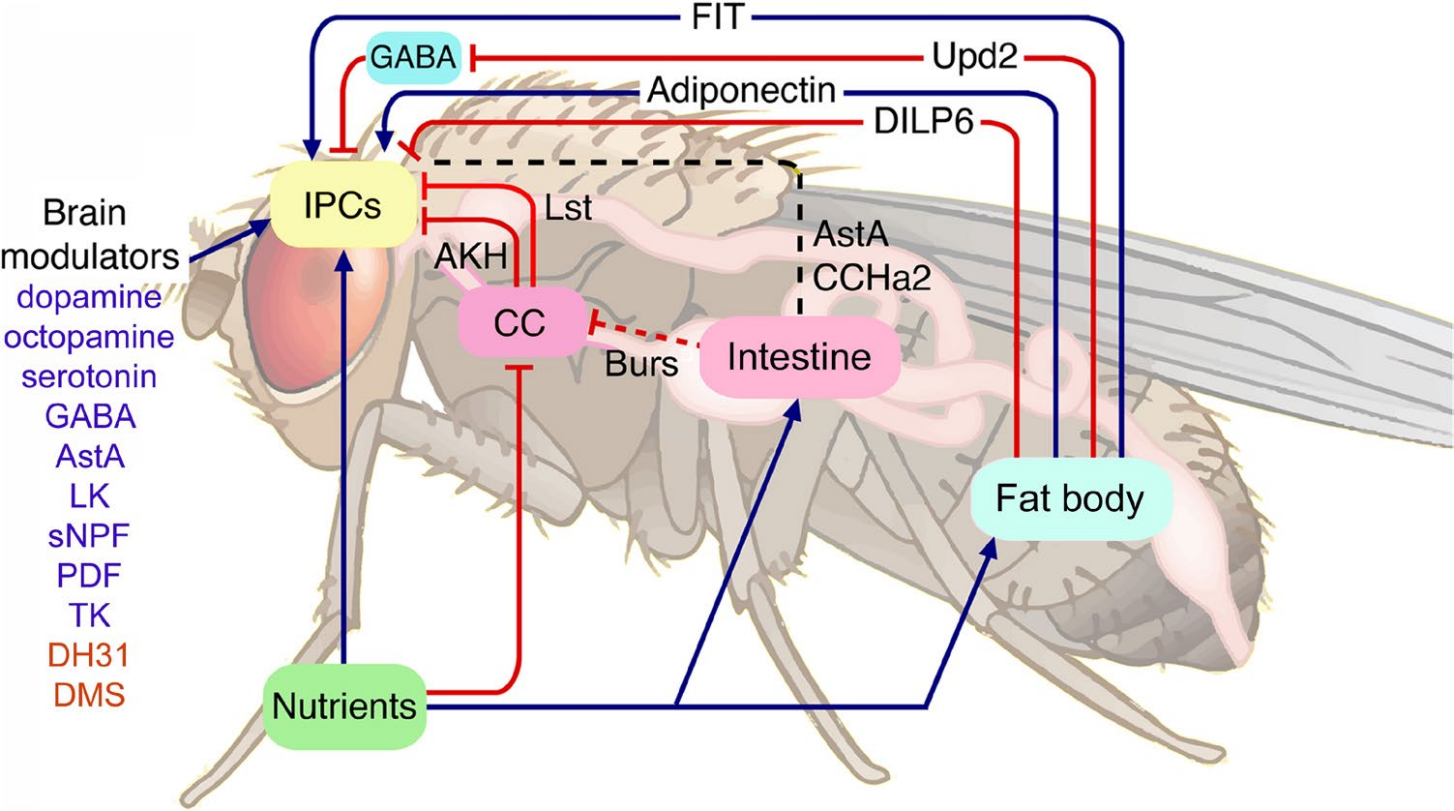
Scheme depicting converging pathways that regulate insulin-producing cells (IPCs) in the adult brain of *Drosophila*. This illustrates how IPCs integrate multiple inputs. Blue arrows depict stimulatory inputs and red bars show inhibitory ones. Dashed black line indicates incompletely known mechanisms. The IPCs are regulated by neurons in the brain releasing serotonin (5-HT), octopamine (OA), dopamine (DA), allatostatin-A (AstA), leucokinin (LK), short neuropeptide F (sNPF), and tachykinin (TK), as well as GABA. In addition, diuretic hormone 31 (DH31) and myosuppressin (DMS) have recently been implicated as IPC modulators (Held et al. 2024). The fat body is nutrient sensing and releases adiponectin-like polypeptide, Upd2, and DILP6 after carbohydrate intake. Upd2 acts (inhibitory) on GABAergic brain neurons and thereby relieves inhibition of the IPCs. Adiponectin and DILP6 act directly on the IPCs. Another factor FIT (female-specific independent of transformer) is a protein-specific signal released from the fat body after a protein meal. The corpora cardiaca (CC), under conditions of low sugar, releases limostatin (Lst) and adipokinetic hormone (AKH) and thereby inhibits release of DILPs. The intestine has nutrient-sensing cells and release peptide hormones into the circulation. Two gut peptides have been shown to act on IPCs, allatostatin A (AstA), and CCHamide2 (CCHa2), whereas bursicon (Burs) from the gut acts on brain neurons, which in turn act on CC to diminish AKH production (dashed line to indicate indirect action via brain). There are other gut peptides that act on the CC or brain neurons that in turn act on IPCs (e.g., DH31 and neuropeptide F; not shown here). This figure is updated from (Nässel and Zandawala 2019), with permission. For further updates on signaling to IPCs, see (Held et al. 2024)

The above examples dealt with divergent signaling of NPHs that converge on specific targets. However, neurons and NSCs that produce NPHs can also receive multiple converging inputs (“integrative connectivity”). For example the insulin-producing cells (IPCs) of the brain are under strict state-dependent regulation by multiple signals, including serotonin, octopamine, dopamine and several NPHs and fat-body derived factors (Held et al. 2024; Nässel and Vanden Broeck 2016). Many are shown in Fig. 9 and are discussed in more detail in (Held et al. 2024; Nässel and Vanden Broeck 2016; Nässel and Zandawala 2020). The IPCs thus constitute a well-modulated hub that ensures metabolic homeostasis under varying external and internal conditions (Held et al. 2024; Liessem et al. 2023).

These are just a few examples of the multiple divergent and convergent actions of NPH. Others can be seen in Table 2 and Fig. 7 or found in some recent reviews (Kim et al. 2017; Nagata and Zhou 2019; Nässel and Zandawala 2019, 2020, 2022). Some general areas that seem especially active in NPH research at present are roles in feeding (including olfaction, taste, appetite, satiety, food search), clock function and circadian activity/sleep, courtship and reproduction, interorgan signaling, metabolism, and osmoregulation. Also roles of NPH signaling in developmental processes and regulation of ecdysone and juvenile hormone are under study but were largely ignored in this review for space reasons.

I end this section with an example of an interesting signal system that involves transfer of a peptide from one insect to another and thus functions as an allomone. This is mediated by the *Drosophila*-specific sex peptide (SP), which is produced in male accessory glands of the reproductive tract and transferred to a female during copulation [see (Kubli 2003; Wolfner 2002)]. SP acts on specific neurons in the female and alters her behavior and physiology for about a week, and the neuronal circuitry underlying the behavior has been mapped (Dickson 2008; Wang et al. 2021; Yapici et al. 2008). SP induces a decreased responsiveness to further mating attempts and alters feeding, metabolism, and sleep pattern as well as an increased egg production and oviposition (Isaac et al. 2010; Okamoto and Watanabe 2022; Yapici et al. 2008).

### Open questions in insect NPH research

1. It is generally accepted that neuropeptides may act at a distance by paracrine (non-synaptic) signaling, also termed volume transmission based on experimental data from mammals [see (Jan and Jan 1983; Taber and Hurley 2014; Zupanc 1996)]. Thus, neuropeptides released within the CNS are largely considered as neuromodulators that affect action of small molecule neurotransmitters (SMNs) at synapses (Bargmann 2012; Hökfelt et al. 2018, 1986; Merighi 2002; Nässel 2009; Nusbaum et al. 2017; Schlegel et al. 2016; Svensson et al. 2019). This concept needs to be explored further in insects where only some indirect evidence is available so far [see e.g., (Hofbauer et al. 2024; Wohl et al. 2023)]. It would be valuable to determine how far NPHs can diffuse within the insect CNS and whether they indeed act non-synaptically at local and/or distant targets.
2. Related to the above is the need for a complete *Drosophila* NPH connectome. In *C. elegans* where a complete synaptic connectome was available already in 1986 (White et al. 1986a) such an analysis is available (Ripoll-Sánchez et al. 2023; Watteyne et al. 2024) and also for the polychaete worm *Platynereis dumerilii* (Williams et al. 2017). Only two such neuronal networks have been explored in *Drosophila*: NSCs (McKim et al. 2024) and clock neurons (Reinhard et al. 2024).
3. The precise distribution of GPCR protein is not known in detail for any NPH in *Drosophila*. We mainly rely on reporter expression from GAL4-drivers and single-cell transcriptomics data, but neither of these inform accurately about receptor protein location.
4. Quite a few NPHs and GPCRs remain understudied. Curiously, the CNS functions of the first sequenced neuropeptide, proctolin, remain unknown. Another, example is the products of the dFMRFa gene (and related peptides in other insects). Although *bona fide* extended FMRFamides have been known in *Drosophila* since 1988, only recently a few studies addressed their functions in feeding and metabolism (Miyamoto et al. 2024; Song et al. 2023; Wu et al. 2024).
5. Peptidergic co-transmission with SMNs is common in brains of mammals (Svensson et al. 2019), but systematic analysis of co-expression of NPHs and SMNs in insect brains is lacking [see (Nässel 2018)]. Single-cell transcriptomics of some neuron populations (clock neurons and NSCs) suggest that such co-expression is extensive also in *Drosophila* (McKim et al. 2024; Reinhard et al. 2024), but needs to be confirmed by in situ hybridization and immunohistochemistry.
6. Several studies (see Table 2) suggest that gut EEC-derived NPHs act on neuronal circuits (or NSCs) in the brain. However, it is not clear whether NPHs can penetrate the blood brain barrier in *Drosophila,* and if so by what mechanism(s).
7. Are there functional consequences of the variation in number of different NPHs existing in different insect species? Today with numerous transcriptome and peptidome studies of various insect species it is clear that some insects, like *Drosophila* has a relatively small number of NPH precursors (about 50) (Nässel and Zandawala 2019), whereas some, like locusts, have a much larger set (about 80) (Ragionieri et al. 2021). Thus, several types of neuropeptides are present in many insect species, but missing in some. In *Drosophila* for instance, several NPHs well represented in other insects are missing, notably adipokinetic hormone/corazonin-related peptide (ACP), allatotropin, calcitonin, elevenin, innotocin, neuroparsins, and thyrotropin releasing hormone [see (Nässel and Zandawala 2019)].
  A study specifically addressing this NPH variability in 31 species of beetles (coleoptera) showed a number of NPHs that are present in some species but not all, others that are commonly found in many insect species, are totally missing in beetles (for instance allatostatin-A, corazonin, LK) (Pandit et al. 2019). Interestingly, this study shows that only four of the studied NPHs are present in all beetle species (allatostatin-B, DH44, insulin-like peptide and ITG) and three in all but one species (neuroparsin, myosuppressin, and NPF). Why are certain neuropeptides missing in some insect species and not in others? It is likely that many NPHs are missing due to gene losses, as the beetle study suggest (Pandit et al. 2019). That study asked whether the phylogenetics, habitat, dietary behavior or the body size of the different beetle species played a role in the complement of NPHs, but found no correlation. In summary, it remains to be investigated whether the lack of a specific neuropeptide means loss of a function, or if another NPH takes over the role of the missing one.
8. Related to the above is the question to what extent the functions of a given NPH are the same in different insect species. Given that most NPHs display pleiotropic functions, it is not likely that all functions are conserved between species, but how much variability is there? Certainly, the molecular structures of NPHs and GPCRs display evolutionarily conservation among bilaterian animals (Elphick et al. 2018; Jékely 2013; Mirabeau and Joly 2013), and in general terms, some functions of several NPHs appear conserved as well. A more systematic analysis is required to reveal the extent of conserved NPH functions between species.
9. The endogenous downstream signaling systems, associated with the GPCRs, such as G proteins and ion channels, and protein kinases coupled to the receptor activity are understudied in insects.
10. NPH signaling acts over specific time scales, commonly slower than SMNs, but still limited by peptidase activity and receptor desensitization. A problem in this context is that most studies of NPH function in *Drosophila* and other insects still utilize crude methods for “transient” activation and inactivation of peptidergic neurons (e.g., optogenetics, thermogenetics, and gene-switch). Furthermore, the stimuli used may be far stronger than during natural activation of neurons. Other types of interference used are chronic (mutants, gene over expression or knockdown, manipulations of membrane excitability, and so on). NPH signaling is likely to be subtler (both temporarily and quantitatively). The question is how close are we to mimicking actual endogenous NPH signaling and can improved methods be devised?

## Concluding remarks

This review summarizes 50 years of NPH research in insects, starting with the early history and ending with a brief account on recent findings. The early years following the sequencing of proctolin in 1975 were spent identifying novel NPHs and mapping their localization to neurons in a number of larger insects, such as moths, cockroaches, locusts, blowflies and kissing bugs and functional studies were restricted to a few NPHs. These were either using endocrinological analysis of a few types of peptide hormones in, e.g., development, lipid metabolism, pheromone production, and diuresis or in vitro assays of visceral muscle contractions and secretion in renal tubules. The years following the genome sequencing in *Drosophila* and a few other invertebrates are harder to summarize due to the drastically increased number of publications on a wide range of novel aspects of NPH signaling. These were, for example, characterization of numerous new NPHs and their cognate receptors (first detected in the genomes), improved techniques for NPH mapping and neuron connectivity, introduction of numerous genetic methods for functional analysis of NPH signaling, and establishment of numerous novel behavioral assays. Analysis of NPH function went from relatively simple assays to systems analysis where neuronal networks, behavior regulation or interorgan signaling were the focus, commonly assayed with advanced genetic techniques, sophisticated imaging, and complex behavior paradigms or clever assay systems. We are now in the era when the synaptic connectome of the whole *Drosophila* brain has been completed (Dorkenwald et al. 2024). However, as noted in this review, the NPHs may require their own “connectome” while they do not necessary act synaptically. Thus, at present, a pairing between peptidergic neurons (senders) and putative target neurons (receivers) can be spotted in the synaptic connectome and the candidate receivers can be assayed for NPH receptors in single-cell transcriptome databases [see (McKim et al. 2024; Reinhard et al. 2024; Ripoll-Sánchez et al. 2023; Watteyne et al. 2024)]. A challenge now is to model state/context-dependent neuronal signaling in networks of the brain where we take into account both synaptic signaling and NPH-mediated neuromodulation.

## Supplementary information

Below is the link to the electronic supplementary material.

Supplementary file1 (PDF 3295 KB)

## Data Availability

No datasets were generated or analysed during the current study.

## Abbreviations

AKH: Adipokinetic hormone
Ast-A: Allatostatin-A
Ast-C: Allatostatin-C
Burs: Bursicon (Burs)
Capa: Capability (CAPA-1 and 2, Capa-pyrokinin)
CCAP: Crustacean cardioactive peptide
CCHa: CCHamide-1 and 2 (CCHa-1 and 2)
CNMa: CNMamide
CRZ: Corazonin
DH31: Diuretic hormone 31
DH44: Diuretic hormone 44
DILP: *Drosophila* Insulin-like peptide (DILP1-8)
DMS: Dromyosuppressin
DSK: Drosulfakinin
EH: Eclosion hormone
ETH: Ecdysis triggering hormone
FMRFa: FMRFamide
Hugin: Hugin-pyrokinin (hug-PK)
ITP: Ion transport peptide
LK: Leucokinin
MIP: Myoinhibitory peptide (Allatostatin-B)
NPF: Neuropeptide F
NPLP1: Neuropeptide-like precursor 1
NTL: Natalisin
sNPF: Short NPF
OK: Orcokinin (OK-A and B)
PBAN: Pigment biosynthesis activating neuropeptide
PDF: Pigment-dispersing factor
Proctolin: Proctolin
PTTH: Prothoracicotropic hormone
SP: Sex peptide
SIFa: SIFamide
TK: Tachykinin
Trissin: Trissin

## Footnotes

1 This review is written in celebration of the 100th anniversary of Cell and Tissue Research (CTR) in 2024. I published my very first scientific paper in this journal in 1975 [Nässel, D.R., 1975. The Organization of the lamina ganglionaris of the prawn, *Pandalus borealis* (Kröyer). *Cell and Tissue Research* 163**,** 445–464.] when I was a PhD student in Lund, Sweden, working on the visual system of crustaceans. Little did I know then that I would still be dabbling with science and writing a review for the same journal 50 years later. I dedicate this review to the memory of the late Professor Andreas Oksche (1926—2017) who was the editor in chief of CTR for many years and with whom I interacted on many occasions. He was an old school German professor (and gentleman) who would address most colleagues with title and last name. Thus, I was thrilled and honored when he sent me a letter (yes, snail mail) in 1988 to congratulate me to being appointed full professor in Stockholm, and he addressed me Dick and signed the letter Andy. CTR was very important for me during my formative years in neuroscience and endocrinology and is still a very valuable journal in my field now that I am going down for landing after more than 50 years in science.
