# Supplementary material for: A brief history of insect neuropeptide and peptide hormone research
Source: Cell Tissue Res. 2024 Dec 10;399(2):129–59. doi: 10.1007/s00441-024-03936-0 (PMC11787221; doi:10.1007/s00441-024-03936-0)
Supplement: Supplementary file 1 — Supplementary file1 (PDF 3295 KB) [file 441_2024_3936_MOESM1_ESM.pdf]

Supplementary material file

A brief history of insect neuropeptide and peptide hormone research

Dick R. Nässel
Department of Zoology, Stockholm University, S-10691 Stockholm, Sweden

Supplementary Fig. 1

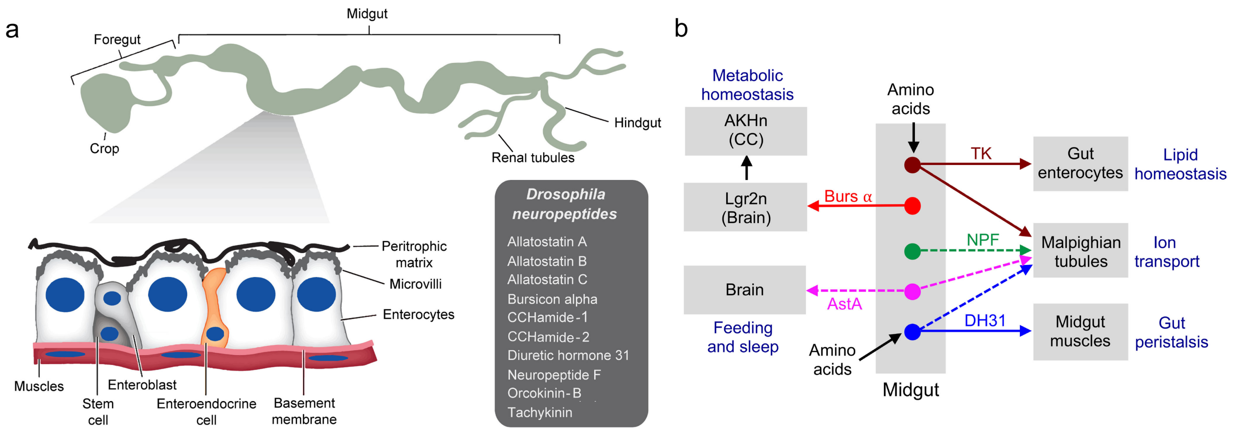

S. Fig. 1. Neuropeptides in enteroendocrine cells (EECs) of the intestine. **a.** A scheme depicting the organization of the digestive tract in adult *Drosophila*. The *Drosophila* gut is comprised of the foregut, midgut, Malpighian (renal) tubules and hindgut. The lower part illustrates that the midgut is composed of five different cell types: muscle cells, stem cells, enteroblasts, enterocytes and enteroendocrine cells. Ten NPHs expressed in the adult *Drosophila* EECs are listed in the box. This figure is from (Nässel and Zandawala, 2019) with permission, which was in turn redrawn from (Lemaitre and Miguel-Aliaga, 2013). **b.** Schematic depiction of signaling from EECs. Solid arrows indicate that experimental data are available for function, and dashed arrows depict hypothetical functions. See text for details, and references. AKHn, AKH producing cells in CC; Lgr2, bursicon receptor; other acronyms in Table 2. This figure is from (Nässel and Zandawala, 2020) with permission.

References

Lemaitre, B., Miguel-Aliaga, I., 2013. The Digestive Tract of Drosophila melanogaster. *Annual Review of Genetics, Vol 47* 47**,** 377-404.

Nässel, D.R., Zandawala, M., 2019. Recent advances in neuropeptide signaling in *Drosophila*, from genes to physiology and behavior. *Progr Neurobiol* 179**,** 101607.

Nässel, D.R., Zandawala, M., 2020. Hormonal axes in *Drosophila*: regulation of hormone release and multiplicity of actions. *Cell Tissue Res* 382**,** 233-266.
